# Implementation science for ambulatory care safety: a novel method to develop context-sensitive interventions to reduce quality gaps in monitoring high-risk patients

**DOI:** 10.1186/s13012-017-0609-5

**Published:** 2017-06-24

**Authors:** Kathryn M. McDonald, George Su, Sarah Lisker, Emily S. Patterson, Urmimala Sarkar

**Affiliations:** 1 University of California Berkeley, School of Public Health, 50 University Hall, Berkeley, 94720 CA USA; 2 Stanford University, Center for Health Policy/Center for Primary Care and Outcomes Research, 117 Encina Commons, Stanford, 94305 CA USA; 3 Department of Medicine, School of Medicine, University of California San Francisco, 1001 Potrero Avenue, San Francisco, 94110 CA USA; 4 Ohio State University, College of Medicine, School of Health and Rehabilitation Sciences, Division of Health Information Management and Systems, 453 W 10th Ave, Columbus, 43210 OH USA

**Keywords:** Organizational interventions, Diagnostic error, Ambulatory care, Cancer, Patient safety, Human factors, Journey mapping, Design seeds, Patient monitoring

## Abstract

**Background:**

Missed evidence-based monitoring in high-risk conditions (e.g., cancer) leads to delayed diagnosis. Current technological solutions fail to close this safety gap. In response, we aim to demonstrate a novel method to identify common vulnerabilities across clinics and generate attributes for context-flexible population-level monitoring solutions for widespread implementation to improve quality.

**Methods:**

Based on interviews with staff in otolaryngology, pulmonary, urology, breast, and gastroenterology clinics at a large urban publicly funded health system, we applied journey mapping to co-develop a visual representation of how patients are monitored for high-risk conditions. Using a National Academies framework and context-sensitivity theory, we identified common systems vulnerabilities and developed preliminary concepts for improving the robustness for monitoring patients with high-risk conditions (“design seeds” for potential solutions). Finally, we conducted a face validity and prioritization assessment of the design seeds with the original interviewees.

**Results:**

We identified five high-risk situations for potentially consequential diagnostic delays arising from suboptimal patient monitoring. All situations related to detection of cancer (head and neck, lung, prostate, breast, and colorectal). With clinic participants we created 5 journey maps, each representing specialty clinic workflow directed at evidence-based monitoring. System vulnerabilities common to the different clinics included challenges with: data systems, communications handoffs, population-level tracking, and patient activities. Clinic staff ranked 13 design seeds (e.g., keep patient list up to date, use triggered notifications) addressing these vulnerabilities. Each design seed has unique evaluation criteria for the usefulness of potential solutions developed from the seed.

**Conclusions:**

We identified and ranked 13 design seeds that characterize situations that clinicians described ‘wake them up at night’, and thus could reduce their anxiety, save time, and improve monitoring of high-risk patients. We anticipate that the design seed approach promotes robust and context-sensitive solutions to safety and quality problems because it provides a human-centered link between the experienced problem and various solutions that can be tested for viability. The study also demonstrates a novel integration of industrial and human factors methods (journey mapping, process tracing and design seeds) linked to implementation theory for use in designing interventions that anticipate and reduce implementation challenges.

**Electronic supplementary material:**

The online version of this article (doi:10.1186/s13012-017-0609-5) contains supplementary material, which is available to authorized users.

## Background

A seminal National Academy of Medicine (NAM) report asserts that most people will experience at least one diagnostic error -- a delayed or inaccurate diagnosis -- in a lifetime, “sometimes with devastating consequences.” [1]. In ambulatory care, one of 20 patients in the United States experiences potentially preventable diagnostic errors annually [2, 3]. Missed cancer diagnoses are the leading reason for paid medical malpractice claims in the ambulatory setting [4, 5].

Widespread research across specialties demonstrates that inadequate monitoring in high-risk outpatients leads to preventable high-risk events and significant patient harm [5, 6]. For example, patients who have a positive fecal blood test but no follow up colonoscopy within a reasonable time period may experience a missed opportunity to detect and successfully treat colon cancer [7, 8]. The National Comprehensive Cancer Network has monitoring guidelines for screening (active and initial) as well as post-treatment cancer recurrence surveillance [7, 9–17]. Proactive and efficient strategies to respond to high-risk situations—such as overdue imaging or blood tests—are urgently needed to reduce the safety gap in evidence-based monitoring for cancer [18–23].

Impoverished populations (those served by “safety-net” settings in the United States) are particularly prone to failures in patient monitoring, given high prevalence of limited health literacy, as well as barriers like lack of transportation, inability to leave work for medical appointments, and a myriad of other obstacles to engaging with the health care system [24–27]. At the same time safety-net health systems often lack critical Health Information Technology (HIT) infrastructure and resources (e.g., personnel time) to devote to monitoring these vulnerable populations [28]. Common software development practices favor mass production and rapid adoption over user-specified customization necessary for long-term sustainability for challenging workflows [29, 30]. To accomplish robust patient monitoring and prevent adverse events, it is critical to identify setting- and population-specific vulnerabilities and needed attributes of effective interventions, whether technical, social, organizational or a combination.

Theories for complex delivery system interventions stress the importance of studying human and contextual aspects of change [31, 32]. The interactions between and within multiple levels of the health care system are posited to affect quality improvement projects [33]. The recent NAM Improving Diagnosis report included a high-level conceptual framework with these factors, among others such as clinical reasoning and teamwork that contribute to diagnostic safety and improvement [1]. The NAM framework explicates that patient and systems outcomes are produced by the diagnostic process which evolves over time, within the context of a larger work system composed of diagnostic team members, tasks, technologies and tools, organizational elements, and the physical environment [34–37]. To reduce the chance of missing a cancer diagnosis, vulnerabilities need to be addressed within both the ambulatory care’s diagnostic process and work system. In other words, what work system factors produce robust monitoring (systems outcomes) and fewer diagnostic errors (patient outcomes)? Research in this area is nascent, with many unknowns about specific vulnerabilities, patient safety intervention opportunities, and subsequent implementations [1, 38]. Taylor et al identified four theoretical domains of contextual features determined by expert consensus as important for patient safety intervention implementations: safety culture, teamwork and leadership involvement; structural organizational characteristics; external factors; and availability of implementation and management tools [39, 40].

To develop theory-based, context-informed organizational interventions for closing the safety gap, our study introduces a unique integration of user experience and human factors methodologies: journey mapping and design seeds. We apply journey mapping to clinician-centered workflow focused on patients at high risk for a missed monitoring opportunity to diagnose cancer [41]. Previous applications have taken the perspective of an individual patient’s journey within a health care setting [42–44]. Design seeds are solution attributes that separate the goal of a modular intervention (e.g., alerting patients that they need to return to the clinic) from the means for achieving it (e.g., use of a web portal messaging system) [45]. They have the advantage of generating multiple solutions to the same problem so unknown vulnerabilities and preferences can be uncovered, interventions can be tailored to different contexts, and more solution variations can be considered to evaluate correct fit [46]. To apply design seeds to patient monitoring,^1^ we draw from a somewhat analogous situation studied outside of health care: intelligence analysts who experience time pressure and data overload as they cull through numerous documents to identify national security threats [47]. Our approach will inform prototyping, piloting and full-scale testing of technical and organizational interventions, with the aim of producing robust population-level monitoring solutions for widespread implementation.

## Methods

### Design

We conducted formative research, following a 6-stage co-development process between the research team and frontline clinicians (attending doctors, residents, nurse practitioners, registered nurses) to identify solution attributes of a comprehensive intervention for more robust monitoring of high-risk cancer conditions over time (Table 1). The research team applied human factors strategies and organizational theory about complex adaptive systems within five specialty clinics to identify vulnerabilities and generate desirable solution attributes for interventions [1, 39, 48, 49].

**Table 1:** Co-Development Research Process

Key Questions Based on NAM Improving Diagnosis Framework PROBLEM: What vulnerabilities exist in monitoring outpatients for high risk conditions (e.g., cancer)? SOLUTION: What elements of work systems and the diagnostic process are important to produce robust monitoring & thereby reduce diagnostic errors?
Stage 1: Identify 5 High-risk Populations and Clinical Informants • Review literature • Corroborate with local clinicians taking care of these patients • Determine with clinical leadership who to interview, based on responsibility for patient monitoring (2-3 clinicians/clinic; 11 total)
Stage 2: Develop Journey Maps • Identify key participants to learn about workflows for each high-risk population • Elicit with semi-structured interview a description of the patient and data flow from worker’s vantage • Visualize this information into swim lanes or “clusters” of activities • Show swim lanes to participants and revise (as needed) • Visit clinic sites to observe critical parts of process (as needed)
Stage 3: Generate Vulnerability List • Abstract vulnerabilities from interview notes and journey maps • Return to clinic participants to validate the list (one or more clinic has indeed experienced vulnerability) • Map validated list of items to theory domains from applicable patient safety frameworks [1, 60]
Stage 4: Analyze Journey Maps for Commonalities • Categorize types of activities in the journey using human factors method of process tracing (novel extension to derive tracings from journey maps) • Generate process trace sequences for each clinic’s workflow [61] • Look for patterns of workflow that are similar and variable across the 5 populations
Stage 5: Develop Design Seeds for Interventions and Link to Implementation Theory • State what a solution would need to do to address vulnerabilities identified from previous stage • Reduce the list to solution attributes (design seeds) that address common problems and needs across clinics • Aim for design seeds that meet the generic needs of robust monitoring and that enable evaluation • Hypothesize which contexts are likely to affect the effectiveness of the implementation of the interventions emanating from the design seeds using Taylor et al’s contextual domains and features (see Additional file 3) [39]
Stage 6: Seek Reactions from Clinics on Design Seeds • Assess anticipated impact (improved monitoring of patients, reduced time spent by clinic team) and relative priority of each design seed (see Additional files 1 and 2 for script and data collection instrument used in each clinic)

### Setting

The San Francisco Health Network is a publicly funded, integrated health network operating under the auspices of San Francisco’s Department of Public Health and includes 14 primary care clinics, urgent care, and specialty care at Zuckerberg San Francisco General Hospital. Last year, there were 539,310 outpatient visits at the hospital alone [50].

The health system serves many of the most medically and socially vulnerable patients in San Francisco. Patients seen at the network’s main clinic and hospital are diverse: 17% are African American, 35% are Latino, 21% are White, and 21% are Asian. Services are provided in over 20 languages. Based on outpatient days, only 1% of the population has commercial insurance, 10% uninsured, 57% Medi-Cal, 21% Medicare, and the remaining 11% covered by other mostly public sources [51]. Others have categorized hospitals according to safety-net burden, with high-burden ranging from 33 or 36% to 100% of patients as those with Medicaid or no coverage [52, 53].

Like many safety-net systems and ambulatory practices, the health system does not have a comprehensive electronic health record system and struggles with information transfer as well as fragmentation of health information across over 50 electronic platforms. Despite some of the HIT challenges and known workarounds typical of these safety-net settings, the organization has a longstanding commitment to both human-centered strategies (patient-centered medical home, plan-do-study-act cycles) and Lean management methods [54].

### Evidence-based Safety Gaps Targeted (Stage 1)

Based on literature about missed and delayed diagnoses, including reports from medical malpractice, we selected five high-risk cancer situations—incidentally-discovered pulmonary nodules, and monitoring for breast, colorectal, prostate, and ear, nose, and throat (ENT) cancers—for which coordination and timely use of data are important for patient safety surveillance but challenging to implement, particularly in safety-net and other low resource settings [4, 5, 55]. These challenging high-risk situations require recurring and timely follow-up care to prevent harm [7, 9–17, 56].

Our team (GS, SL, KM) conducted a series of theoretically informed semi-structured interviews with participants from each of five specialty clinics responsible for these high-risk patients: pulmonary medicine, breast cancer, gastroenterology, urology and otolaryngology. As part of these interviews, we corroborated the specific safety targets by asking frontline clinicians: “What keeps you up at night?” and “What are your clinical hunches about who might fall through the cracks?” Although providers talked about the types of patients lost to follow-up, none of the clinics were enabled with a standardized and efficient method for quantifying how many patients were lost to follow-up, why patients were lost to follow-up, or even which patients were lost to follow-up. Many other health networks share similar struggles with incomplete documentation and measuring the real-time scope of patient safety problems [57].

### Mapping and Analyzing Clinical Workflows (Stage 2 through 4)

The interviews in each of the five cancer clinical settings followed a user-centered design approach called journey mapping, a tool widely used across multiple industries [41, 58, 59]. Journey mapping derives from user experience initiatives in industry that informed the framework proposed in the recent NAM Improving Diagnosis report [1, 35, 60]. The method articulates and documents a process through a specific point of view (typically, a customer). In the health care field, it has been applied to elicit individual patient journeys through the clinical workflow [43]. Our team-based variation of journey mapping has a patient population management view. We elicit specialty care management through the experiences of the clinical team as they try to track the host of patient data required to monitor their high-risk population. To our knowledge, this technique has seldom been applied to the ambulatory setting, and has not been targeted to clinic workflow efficiency or patient safety intervention development.

We directed these journey mapping sessions to: (1) isolate the steps in the patient monitoring journey that are the most critical, time-intensive, and risky relative to the safety gap, (2) identify critical data elements needed to effectively and safely monitor patients, and (3) gather potential attributes of organizational and technical interventions to ameliorate workflow problems. To construct the journey maps, investigators (GS, SL, KM) probed clinical participants with questions such as: “What are you working on?” to elicit actions taken; “Who is responsible for which task?” to learn about monitoring-related activities; “Are there external stakeholders?” and “How important are they?” to surface coordination challenges outside of the clinic. Based on what participants articulated, we constructed a journey map for each clinical pathway with their review and endorsement. The maps focus on the transfer of patient data throughout the patient’s monitoring experience, starting with the initial diagnostic assessment and ending with the ongoing follow-up. Whenever participants verbalized elements of the pathway that were particularly vulnerable to error or poor monitoring, we marked the activity with a bull’s eye target, also referred to by clinicians as a ‘pain point’.

From the journey mapping sessions, we listed all of the vulnerabilities experienced by at least one clinic. To verify the list and gauge how many of the clinics experienced each of the vulnerabilities, we returned to the clinic with a data collection instrument (Additional files 1 and 2). We also mapped the vulnerabilities corroborated by at least one clinic to domains from patient safety theoretical frameworks [1, 60].

Using standard process tracing techniques from human factors, we categorized and summarized the sequence of activities described in journey maps [61]. The trace sequences are used to determine the similarity of activity flow among clinics that monitor high-risk populations as well as any differences between clinics to inform well-designed interventions.

### Developing Design Seeds and Linking to Implementation Theory (Stages 5)

**Fig. 1:**
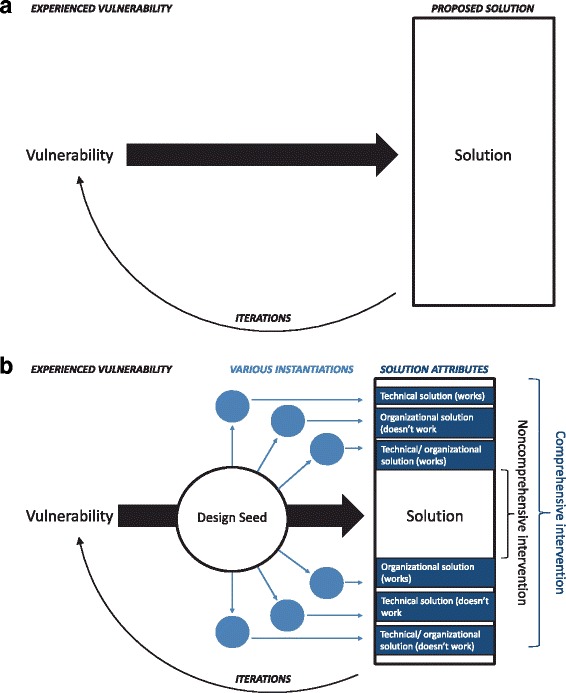
**a** Technical Intervention Development Cycle. **b** Socio-Technical Intervention Development Cycle: Design Seed Theory. The figures show that the socio-technical design seed intervention development adds an intermediate step that translates expressed vulnerabilities into multiple solution possibilities and evaluation markers. In contrast to a singular solution provided when linking a problem directly to a proposed solution, design seeds tease apart the expressed vulnerability to offer a distinct set of evaluable solutions that can be tested independently. Design seeds yield a menu of modular options for implementers considering differing organizational context

Design seeds and the human factors approaches from which they stem have been used outside of health care for development of complex socio-technical interventions [47, 62]. They serve as bridges to technical and organizational solution options that can be designed differently depending upon context, but that use common attributes. As such, they offer an appealing addition to the implementation science toolkit. In simple terms this approach replaces the typical technical approach (Fig. 1a) with a theoretically based socio-technical system understanding (Fig. 1b). As shown in Fig. 1b, design seeds link the vulnerabilities experienced to potential solutions in a specific and evaluable way. This promotes the evaluation of a “seed” to a solution, rather than a full-fledged solution itself as is practiced in software development cycles often used in HIT [63]. By jumping directly from “problem” to “solution,” one opens the door to various misdirected applications that do not appropriately mediate the diverse instantiations of a problem. The evaluation of design seeds prior to the development of a solution creates an opportunity for more cost-effective and user-customized solutions [47].

Since a design seed features a series of evaluable statements, the approach enables intervention testing at the right point in the pathway for a specific action (e.g., does the intervention work according to the design seed criteria? yes, no, or partially). In order to set up theory-based implementation, we (KM, SL) independently used these statements to hypothesize which contexts are likely to affect the effectiveness of implementation of the interventions emanating from the design seeds. We used Taylor et al’s four contextual domains and 13 specific features that a technical expert panel judged as high priority for assessment (as opposed to simple description) in the evaluation of a varied range of patient safety intervention implementations (Additional file 3) [39].

### Assessing Clinician Reactions to Design Seeds (Stage 6)

To gain insight about the importance of the design seeds, we developed and tested a data collection script and instrument (Additional files 1 and 2). We then returned to the frontline clinician participants from each clinic, who reviewed each design seed, assessed likelihood of improved monitoring and likelihood of reducing time spent monitoring, and ranked the set of seeds for relative overall importance.

## Results

From January 2015 to February 2016, we convened one or more journey mapping sessions with clinicians and staff at five specialty clinics to establish the workflow for monitoring high-risk patients. As expected, all clinics participate in teaching alongside patient care, have similar patient demographics with accompanying operational challenges (e.g., translation services, transportation needs), and use the same underlying electronic health record system but work within a larger system of fragmented record-keeping systems (e.g. different specialty-specific EHRs, electronic systems restricted to on-site devices, paper-based systems). The mapping process also revealed variability in organizational approaches to monitoring high-risk patients, including the types of personnel involved in various monitoring-related tasks (e.g. resident versus nurse responsibility for tracking) and the specific steps taken to monitor high-risk patients (e.g., use of notebook-based list of patients versus lack of a structured tracking tool).

### Journey Maps: How Specialty Clinics Monitor High-Risk Patients

**Fig. 2:**
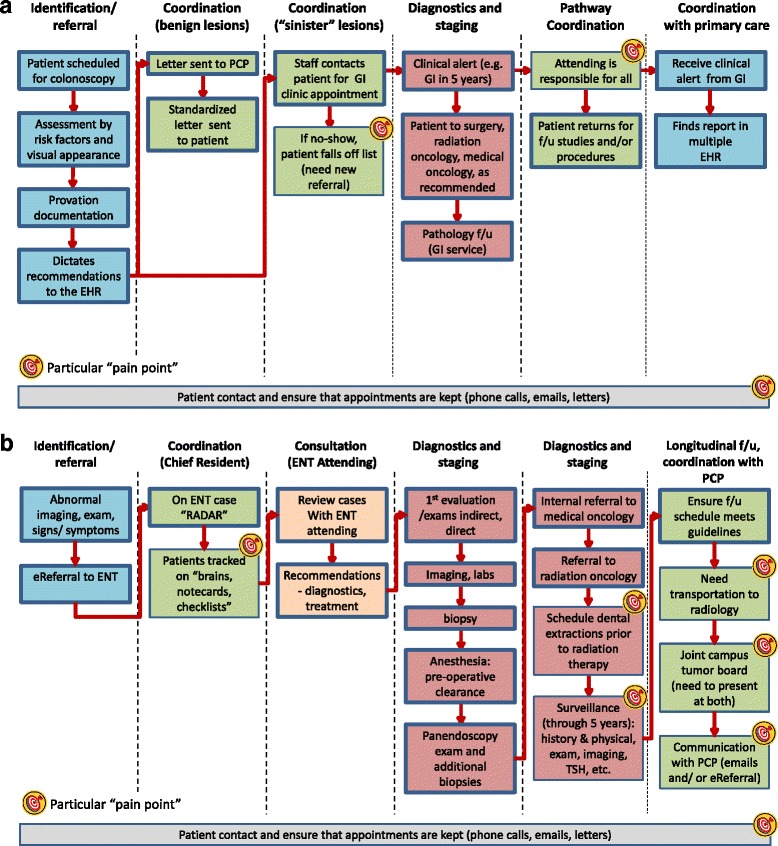
**a** Abnormal Colonoscopy Journey Map. **b** Ear Nose and Throat (ENT) Cancer Journey Map. Investigators constructed journey maps that follow the management of patients who are being monitored and treated for cancer, as articulated by clinical participants. Similar activities and actions are clustered into vertical “swim lanes,” *arrows* indicate the flow between actions, and bull’s eye targets mark actions that are particularly vulnerable to missed monitoring. For example, an action that does not have an “owner” may instigate a higher risk for patient loss to follow-up

For each clinic, we constructed a journey map as shown in Fig. 2a, the abnormal colonoscopy workflow, and Fig. 2b, the ENT cancer workflow. These journey maps follow the management of patients with concerning conditions requiring cancer surveillance, diagnosis, monitoring and treatment, starting with referral to the respective subspecialty clinic. Each journey map contains swim lanes (visual columns) to group similar activities, flow arrows to represent patient and information movement, and targets to highlight areas of vulnerability for monitoring as expressed by clinic personnel.

For example, an abnormal colonoscopy triggers entry into the gastroenterology clinic workflow (Fig. 2a), which is adjudicated by the attending doctor. The first swim lane clusters the activities related to referrals. The next two swim lanes separate two different levels of diagnostic concern and coordination – one for benign lesions which just require notifying the primary care doctor, and the other for “sinister” lesions which precipitate a series of actions within the specialty clinic, as well as coordination with others based on subsequent findings (e.g., pathology, primary care, oncology). The bull’s eye target on the box -- “if no-show, patient falls off the list” -- means that the clinic is aware of this vulnerability, but does not have any further, regular steps to reduce the risk of losing a patient to follow-up. The bottom of the diagram illustrates that patient-related contacting happens throughout the workflow; the associated target conveys the challenges in reaching patients outside of clinic and assuring that they can make it to follow-up encounters.

The ENT clinic (Fig. 2b) reported similar challenges contacting patients monitored and treated for cancer, as did all other clinics (Additional file 4). The activities performed by the ENT clinic for cancer monitoring cluster into four swim lanes—case identification and referral, coordination, consultation, and care pathway. In this clinic, the coordination activities do not follow from a particular clinical scenario (the benign versus sinister lesion), but instead relate to a particular role, the chief resident. As a result, this clinic identified four separate vulnerabilities related to the busy chief resident’s responsibility to keep patients on the “ENT Radar” without any specific tools besides paper notecards, while also coordinating resources such as transportation for patients, tumor board presentations, and communication of follow-up requirements to primary care providers (PCPs). The care pathway swim lanes sketch out a series of diagnostic activities and pre-treatment preparation. The last stage of this clinic’s care pathway is patient surveillance after treatment. No specific responsibility assignment exists for patients who require regular surveillance to monitor for cancer recurrence, so the ongoing surveillance activity box is labeled with a bull’s eye target, indicating another vulnerability.

The journey maps intentionally tell only part of the story as they represent the journey told from a single clinic’s perspective. For example, all of the subspecialty clinics have interactions with PCPs, but only when an individual clinic spoke about dependencies on the PCP for the patients that they monitor did we include the PCP in a journey map. For incidental lung nodules, breast cancer oncology navigation service, and abnormal colonoscopies, the specialty clinics rely on the PCP to remind patients to follow-up at the necessary intervals since they have minimal contact with these patients.

### Challenges Experienced in the Clinics

Based on the interview notes and journey maps from all five clinics we developed a comprehensive list of vulnerabilities described by at least one clinic (see Table 2). We identified 45 distinct vulnerabilities, and mapped these to domains from patient safety theoretical frameworks: 36 relate to work system factors that are inherent to environment, task, technology, organization and people, while 9 vulnerabilities correspond to process factors that reflect interactions between people or with systems [1, 60]. Each clinic reviewed the list at least four months after journey mapping to validate high-priority vulnerabilities that persist over time despite ongoing organizational changes and to record differences between clinics. Only two vulnerabilities—1) have to track some patients in own mind or side system, and 2) creating list of patient requiring monitoring takes time – were experienced by all five clinics. At least two clinics (in varying combinations) experienced most of the vulnerabilities. Four of the five clinics verified multiple problems related to the time expended on tasks related to monitoring. The breast cancer clinic experienced only 7% of the full list of possible vulnerabilities, while the others experienced 12% to 34%. This lighter vulnerability burden is perhaps because the breast clinic has separate philanthropic funding that supports patient navigation services, referred to by a participant as a “human tracking system”.

**Table 2:** Vulnerabilities Experienced by Each Clinic

Vulnerability from Specialty Clinician Perspective	# of Clinics Experiencing	Clinic (X = experienced)				
Classified by Framework Domain^a^		B	P	GI	E	U
Work System: Task						
Have to track some patients in own mind or side system	5	X	X	X	X	X
Creating list of patients requiring monitoring takes time	5	X	X	X	X	X
Looking up each patient’s information takes time	4		X	X	X	X
Maintaining list of patients requiring monitoring takes time	4		X	X	X	X
Outside of visit-based care, don’t always know when patients need follow-up monitoring	4	X	X		X	X
Manually monitoring patients is time intensive	4		X	X	X	X
Don’t always know which patients need to be called back for monitoring	3		X		X	X
Have to spend too much time scheduling	2		X		X	
Manually monitoring patients is error-prone	2				X	X
Work System: Technology and Tools						
Analyzing data in ad hoc manner is time intensive	4		X	X	X	X
Inefficient system to create personal, siloed reminders for follow-up	4		X	X	X	X
List of patients we use outdates quickly	3			X	X	X
Can’t divert alerts to other providers	3		X	X	X	
Analyzing data in ad hoc manner is error-prone	3		X		X	X
Don’t always know when patient data is missing	2		X		X	
Can’t find missing data from outside clinic	1				X	
Don’t always want alert when patient status changes	1					X
Don’t have adequate real-time data	1				X	
Can’t edit patient’s care pathway as needed based on frontline data	1		X			
Can’t find missing data within clinic	1				X	
Work System: Organization						
Systems don’t talk to each other	4		X	X	X	X
Don’t have a system that puts patients into subgroups for more efficient monitoring	4		X	X	X	X
Can’t share patient list with entire care team	3		X		X	X
Don’t always have the time to perform the assigned role	2		X		X	
Hard to stratify patients into subgroups for monitoring due to many individual patient differences	2		X		X	
Care plan is poorly documented	2		X		X	
Don’t know what types of scheduling challenges occur most often	1				X	
Work System: People						
Overlapping efforts	4	X		X	X	X
Don’t always know when the loop closes	3			X	X	X
Everyone inputs data differently	2		X		X	
Knowing who is managing at each stage is unclear	2				X	X
Mapping patient to care plan requires clinical judgment	2		X		X	
Work System: Environment						
Coordinating scheduling efforts across care teams is difficult	3		X		X	X
Little or no performance data about monitoring so don’t know where to focus any improvement efforts	3	X	X		X	
Stretched for resources to reach out to all patients in need of follow-up	3		X	X	X	
Unaware of clinic’s performance in patient monitoring	2	X			X	
Process: System-Patient Interaction						
Don’t know when patient misses appointment	4		X	X	X	X
Don’t always know when patient doesn’t have PCP	4	X	X		X	X
Don’t always know patient’s vulnerabilities relevant to monitoring (e.g. patient’s work schedule, can’t get to clinic, substance abuse)	3		X		X	X
Difficulty communicating patient needs with entire care team	2		X		X	
Don’t know when patient changes status	2		X		X	
Process: System-Provider Interaction						
Inconsistent process for informing PCP	3		X		X	X
Can’t use patient data for operational improvement	2		X		X	
Involving PCP when not necessary	1	X				
Process: Patient-Provider Interaction						
PCP doesn’t have overview of all patient info/care pathway	3		X		X	X

^a^Adapted from the National Academy of Medicine Improving Diagnosis Framework, 2015 and Sarkar et al’s System-related Factors, 2014 to classify each reported vulnerability into Work System versus Process, as well as subdomains of these two framework categories [1, 60]

Legend: Clinics designated as B = Breast, P = Pulmonary, G = GI, E = Ear Nose and Throat, U = Urology

Several broad work system challenges emerged from the clinic visits:

- Organized for visit-based care (as opposed to patient management over time)
- Rotating care providers from visit-to-visit due to being a teaching environment (as opposed to having doctors with long-term organizational know-how)
- Lack of clear ownership for the monitoring-over-time function (as opposed to task responsibility and adequate time allocated for this population management function)
- No aggregated real-time lists of those who require follow-up monitoring (as opposed to supportive tools)
- Lack of systematic and transparent approach to patient’s care plan (as opposed to widely known and specified benchmarks and timing for monitoring follow-ups)
- Substantial time pressure limits frontline attention to learning from missed monitoring incidents (as opposed to efforts to analyze data about misses, understand vulnerabilities and develop organization-wide solutions)

This work environment analysis that utilizes the NAM framework underscores the lack of infrastructure and processes organized to support population-level tracking of patients undergoing diagnosis of initial cancer, progressing cancer or recurring cancer. One noteworthy finding was the lack of population-level descriptions of the different types of monitoring care pathways commonly used within a given clinic. For example, the urology clinic participants – an attending doctor, a resident and a nurse – described a composition book where the resident logs all urologic patients who had a pathology result. The composition book is a starting point for population-level tracking of those who are at some risk for being lost to follow-up despite likelihood of needing it. However, the list is not sub-divided or categorized based on findings, conditions, anticipated follow-up pathway (e.g., testing, timing of next visit). The clinic participants noted that they preferred a system to monitor for all urologic cancers rather than restricting to prostate cancer monitoring (journey map focus) and that the composition book re-emerged as a workaround after a technical monitoring system was unsuccessful.

### Process Trace Sequences: Four Critical Activities for Monitoring High-Risk Patients

To simplify the journey maps and enable pattern recognition across clinics (see Additional file 5 for color-coded journey maps), we categorized each action into one of three functional clusters:

- Communicate/coordinate
- Patient activity (contact patient, patient shows up)
- Review or enter data/data systems

**Fig. 3:**
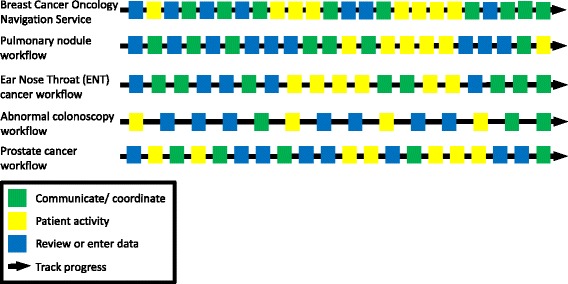
Process Trace Sequences. The display shows process trace sequences of major activities, and the constant tracking to monitor high-risk patients. Each clinic sequence is derived from a tricolor-coded version of its original journey map (Additional file 5).

Figure 3 shows the resultant process trace sequences derived from the journey maps for each of the five clinics. The workflows have similar patterns: review and entering data at the beginning of the journey; a couple activities to communicate and coordinate within the clinic team before seeing a patient, a series of tests and appointments where the patient has to show up, and some patient contact outside the appointments punctuating the middle of the journey; and more communication or coordination actions marking the end of the journey. As noted in the thematic analysis, a fourth critical activity weaves through the sequence:

- Track progress related to patients and their follow-up needs

### Design Seeds: Elements of a Comprehensive and Adaptable Intervention to Save Lives and Time

**Fig. 4:**
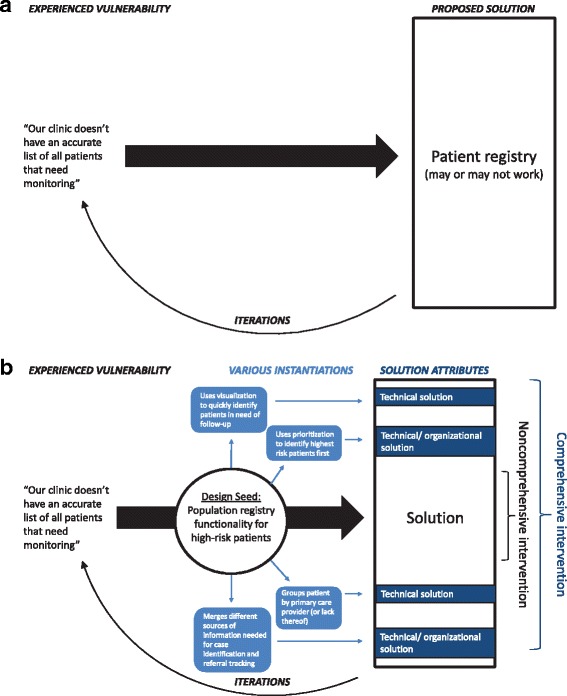
**a** Technical Intervention Development Cycle: Example. **b** Socio-Technical Intervention Development Cycle: Design Seed Example. The Pivotal Role of Design Seeds for Intervention Development: Design seeds offer an important bridge between identifying problems and solutions. For the design seed– a population registry for high-risk patients – the diagram shows the evaluable components of the design seed in the *light blue boxes*. At the solution development stage, different technical and/or organizational interventions can be tested to see if they meet the design seed requirement. Solution components, shown in *dark blue boxes*, that meet the design seed requirement (based on iterative testing) can be assembled as a comprehensive intervention for further testing and deployment

To inform intervention development, we looked for leverage points to alleviate the vulnerability areas that held the highest consequence for failure. We generated a list of 13 leverage points, called design seeds, which correspond to the critical activities for robust patient monitoring, as shown in Table 3. One of the clinics, urology, told us that they had a registry but it was not used. This situation exemplifies the typical solutions pathway, as shown in Fig. 4a. In contrast, based on socio-technical theory, stating simply that a registry “is needed” is too minimalistic and fails to take the organizational context and its potential variations into account. Figure 4b provides an example of the design seed description for functions needed in a population registry of high-risk patients requiring monitoring. The design seed communicates the intent behind the recommendation resulting in a modular - therefore more evaluable - set of solution attributes. Each of the four functions (e.g., groups patients by PCP) shown can support different components of an intervention. In addition, each design seed functional statement can easily be converted into an evaluation question, such as “does the intervention use data visualization in a way that enables rapid identification of patients in need of follow-up?” or “does the intervention allow our clinic to prioritize work in a way that assures that the highest risk patients receive follow-up first?” (Additional file 1 has the detailed functional descriptions for each of the design seeds, as presented to the clinics for feedback.) These descriptions also support hypothesis-generation about contextual features that may have variable effects on whether the intervention is able to achieve its intended design goals(Table 3) (Additional file 3 shows our hypothesized relationships between context features and design seeds).

**Table 3:** Design Seeds Relationship to Critical Activity Categories and Implementation Context

Critical activity category	Design seed	Relevant Context Domains [39]			
		Safety Culture, Teamwork, Leadership	Structural Organizational Characteristics	External Factors	Implementation/Management Tools
Communicate/coordinate	Ability to control data access	X	X	X	X
	Scheduling functionality	X	X		X
	Assign roles and responsibilities	X	X		X
	Triggered notifications	X	X	X	X
Patient activity	Patient support	X	X	X	X
	Complete patient information	X	X		X
Review or enter data	Keeps list up-to-date	X	X		X
	Standardized data entry			X	X
	Complete data capture		X	X	X
	Performance data	X	X	X	X
Track progress	Population registry functionality for high-risk patients	X			X
	Figure out what patients are “on the list”	X	X	X	X
	Customize the patient list	X	X		X

Legend: Design seeds correspond to the four critical activities performed by clinics. To maximize effectiveness in diverse and dynamic settings, designed interventions are considered within the context of a larger work system, split into four major domains by Taylor et al. Hypothesized relationships between context features (e.g., leadership at unit level, local tailoring of intervention) within the four context domains and each design seed are shown in Additional file 3

When clinic participants were presented the preliminary findings from this formative research, they were asked to prioritize the design seeds and suggest revisions of the design seed descriptions. Twelve of the 13 design seeds received ranking in the top 5 in at least one clinic (Table 4). Although the design seed for scheduling functionality did not receive a top 5 ranking, four clinics ranked it seventh, right in the middle of the list, so it was hardly a low priority. The design seed for keeping the list of patients who need monitoring up-to-date received top three or better ranking at four clinics. Three other design seeds – triggered notifications, customize the patient list, and population registry functionality – were ranked in the top 5 by three or more clinics. As noted by one participant, the high priority seeds or solution attributes “were those that bring the right information to the right person at the right time.” Some design seeds had higher salience for only one clinic. Complete data capture, for example, is more problematic for clinics whose patients receive some of their care at other institutions that use other record-keeping systems and EHRs. In these instances, patient data is either inaccessible or must be faxed between sites.

Based on average ratings shown in Table 4, as well as individual clinic ratings, the design seeds received agreement that they would improve monitoring and save time in most clinics. Only one design seed (assigning roles) received disagreement for improving monitoring and reducing time in one clinic because all monitoring is performed by a “one-woman show” (a registered nurse). All design seeds except one (patient support) received the most favorable rating (5) for time saved by at least one clinic. No clinic provided very strong agreement (5) that the design seed for performance data would improve monitoring, although representatives of three clinics (breast, GI and urology) agreed that it would improve monitoring (ratings of 4 on 5-point scale). As one respondent noted, “the scope of the problem would be good to know, but secondary to other needs.” This view is consistent with other studies showing frontline concern that monitored activities will be artificially prioritized over core clinical work [64]. Design seeds viewed as having higher impact potential for saving time and improving monitoring were generally ranked closer to the top by more clinics.

**Table 4:** Importance Ranking of Design Seeds from Five Specialty Clinics

Design Seed	Ranked in Top 5	Rank (Avg)	Improved Monitoring (Avg)	Reduce Time Spent (Avg)
Keeps list up-to-date	P, G, E, U	3.4	4.6	4.8
Triggered notifications	B, G, E	4.2	4.8	4.8
Customize the patient list	B, P, G, U	5.2	4.2	4.6
Ability to control data access	E, U	6.2	4.4	4.2
Population registry functionality for high-risk patients	P, E, U	6.6	4.4	4.2
Complete patient information	G, E	7.2	4.6	4.6
Standardized data entry	G	7.2	4.2	4.4
Performance data	B	7.2	3.6	3.8
Patient support	B, P	7.8	4.2	3.6
Complete data capture	B	8	3.8	4.2
Scheduling functionality	-	8.4	4	4
Figure out what patients are “on the list”	P	9.8	4.2	4.2
Assign roles and responsibilities	U	9.8	3.4	3.6

Legend: Clinics designated as B = Breast, P = Pulmonary, G = GI, E = Ear Nose and Throat, U = Urology

## Discussion

This research highlights the unique and innovative integrated application of methods drawn from human factors engineering (design seeds, process tracing analysis) and user experience studies (journey mapping) to derive context-sensitive and theory-based interventions at the local level. Such focused and potentially scalable work is particularly needed for patients who may be lost to follow-up in systems that are stretched for dollars and time. This project focused on high-risk patients, both clinically due to a potentially concerning finding during an outpatient visit, and due to challenges from a socio-demographic viewpoint. When a patient has a warning signal for a serious condition that has yet to materialize, but may in the future, the ability of a clinical team to watch the patient closely over time hinges on incredible vigilance on the part of individual clinicians - hardly an ideal solution.

These challenges mirror those reported in other health settings with incomplete documentation and limited knowledge of the magnitude of patient safety problems [57]. Providers will often create informal workarounds in response to the lack of comprehensive and coordinated record-keeping systems, which can result in errors as well as redundant efforts [65, 66]. Accompanied by an understanding of these workarounds, resource strapped settings offer a unique opportunity to apply user-centered approaches to redesign socio-technical strategies by integrating user and client needs, the possibilities of technology, and requirements for economic viability [67].

Through mapping how patients are currently monitored for specific high-risk conditions according to evidence-based practice in five specialty clinics in our safety-net setting, we identified 45 different vulnerabilities. Repeatedly, we heard that clinicians worry about properly tracking these patients, and are troubled by the significant personnel time required in carrying out patient-level monitoring activities without tools and organizational approaches for population-level monitoring. In addition, no ongoing performance data currently exists related to the frequency of missed opportunities to monitor these high-risk patients, though efforts are underway [27].

To ameliorate the difficulties identified, we worked iteratively with the clinics to develop the basis for a sound approach to population management of diagnostically high-risk patients. We adapted the journey mapping technique to capture activities and experiences of the clinic team as they manage cohorts of such patients, focusing on the clinician’s monitoring journey. Previous applications have focused on patient journeys and experiences. While each clinic had a different journey map, all teams carried out the same four basic functions with some variation in sequencing and specifics. For example, one function, ‘patient activities’, includes scheduling the patient, assisting patients with barriers to making it to a critical test, seeing the patient when they come into the clinic, conducting an imaging study, and so forth.

Once we understood the clinic teams’ many concerns, particularly the time implications of the current monitoring workload, as well as the potential for errors, we did not jump straight to solutions. The use of design seeds, as a bridge between problems and effective organizational interventions, offers three advantages to leaping over this step. First, design seeds are simple descriptions that state what a solution needs to do, and can be described in a way that allows validation by the users, those on the frontlines at the clinics. For example, clinicians can easily imagine scenarios where patients might not be monitored according to evidence-based guidelines because of ambiguity in who is responsible for tracking high-risk situations (i.e., addressed by the design seed for assigning roles and responsibilities between primary care practitioner and specialist for a patient flagged for further follow-up). Second, design seeds can be supplied to other clinics to learn whether they have face validity outside of this particular safety-net setting. Design seeds support flexibility and tailoring to context, a critical feature for effective implementation of patient safety interventions in different settings [39, 68]. Other clinics could use the feedback exercise to determine whether the 13 design seeds are perceived to improve monitoring and save time in their setting prior to investing in a solution. As a result, one organization could implement and test interventions based on one set of design seeds (e.g., #3, 5, and 7), while another might choose another set (e.g., #2, 3, 4, 6) based on differing contextual enablers, barriers and interactions [33, 37, 39, 40]. Third, design seeds are, by definition, an assessment tool during testing of potential solutions. Does the solution do what the design seed prescribed? Some of the design seeds may result in primarily HIT solutions (triggered notifications), while others may need significant organizational changes (patient support). But most, if not all, will likely require both technical and organizational change.

The use of design seeds, previously applied for complex cognitively rich tasks outside of health care, is adaptive to any organizational setting coordinating layers of cognitively taxing activities meant to accomplish a particular organizational goal [69, 70]. Health networks fragmented by technology, location, and organizational elements are ripe environments for the design seed method as it captures differences in context while moving towards a cohesive end-goal: a solution that works across settings while also targeting specific needs to provide high value to local settings. In our case, we sought to use journey mapping coupled with process tracing and design seeds to identify features of population management interventions for high-risk conditions and treatments to reduce diagnostic error. The flexible structure of these tools, anchored to touch points with end users, enable a generalizable strategy for identifying leverage points, reducing diagnostic delays related to suboptimal monitoring, and increasing organizational effectiveness.

### Limitations

While designed for adaptability across systems, our proposed strategy for developing design seeds would be strengthened by further assessment within other health care systems. At this stage, we know from testing in multiple specialty settings that common themes and variations exist. While each of the clinics in this study has its own leadership, electronic and paper-based systems, and organizational design, we showed that journey mapping paired with process tracing captured both differences and similarities across five settings.

Because data collection was part of quality improvement work in a low resource setting, we relied on a small number of informants from each clinic. An additional limitation is that our design seeds have not received feedback from stakeholders outside the specialty clinic workforce (e.g. patients, information technology providers, caregivers). Any vulnerability tied to patient knowledge or preferences would best be elicited from patients directly, and most likely generate refinement of the patient support design seed. By focusing on the “holders” of the patient data – those stakeholders that most frequently engage with, and bear responsibility for, patient monitoring activities – we have established a foundation from which to build. The approach used fosters an iterative process for data collection that will loop in other stakeholders. Our adaptation of journey mapping and design seeds summarizes a broad, but possibly incomplete, list of activities related to patient monitoring when approached from a cohort perspective.

### Future Work

We sought to draw from organizational analysis used outside of the healthcare setting to inform a practical and scalable intervention geared to reduce missed and delayed diagnosis in high-risk patient populations. Ideally, this approach would be replicated in other specialty areas and sites, including those that are better resourced. We will translate the validated design seeds into a prioritized list of solution attributes to use in development and evaluation of socio-technical interventions. During the organizational change process, we intend to continually reference and iterate journey maps. One of the design seeds – figure out what patients are “on the list” – will require work within the clinics as well as literature reviews targeted to trigger algorithms for identifying patients in need of close, but not urgent, follow-up during their diagnostic journeys [71–73]. We anticipate that interventions evaluated against user needs that are generated with intention and context will be more sustainable, user-friendly, and implemented more successfully than those generated without this human factors approach.

As a nascent area of research, strategies to close gaps in diagnostic safety built from the ground up, as in this study, will first be followed by pilot testing, and ultimately full-scale implementation evaluations with additional measures related to the people (patient, provider), organizational, technology and structural factors predicting desired implementation outcomes [74]. The NAM Improving Diagnosis framework shares a similar multi-level structure with those of implementation science, anticipating future research to improving diagnostic care in an organizationally effective and sustainable way.

## Conclusions

We carried out a multi-stage research process with specialty clinics at an urban publicly funded health system to address an important evidence-based safety gap in ambulatory care: potentially preventable and consequential diagnostic and monitoring delays. Based on surfacing a large number of common vulnerabilities among the clinics, we specified and validated key attributes for a robust socio-technical approach to improving outpatient monitoring that is geared to enable context-sensitive implementation, utilizing industrial and human factors methods linked to implementation theory.

## Additional files

Additional file 1: Script for data collection on design seeds. (DOCX 19 kb)

Additional file 2: Feedback form for data collection on design seeds. (PPTX 135 kb)

Additional file 3: Hypothesized relationships between context features within context domains and each design seed. (DOCX 25 kb)

Additional file 4: Additional workflows (breast, gastroenterology, urology) with targets. (PPTX 108 kb)

Additional file 5: All workflows color-coded as foundation for process trace sequences. (PPTX 109 kb)

## Abbreviations

AHRQ: Agency for Healthcare Research and Quality
EHR: electronic health record
ENT: ear nose and throat
f/u: follow-up
GI: gastroenterology
HIT: Health Information Technology
NAM: National Academy of Medicine
PCP: primary care provider

## Footnotes

1 Patient monitoring for cancer, in this paper, is broadly construed to include an expansive set of diagnostic opportunities, not just one definitive and staged cancer diagnosis. Ambulatory safety risk in this context includes identification of high-risk patients, pre-diagnosis testing, definitive diagnostic procedures (e.g., biopsies), and even longitudinal post-diagnosis follow up (e.g., keeping track of patients for whom treatment is delayed on purpose, or following patients after treatment for cancer recurrence).
