# Supplementary material for: Implementation science for ambulatory care safety: a novel method to develop context-sensitive interventions to reduce quality gaps in monitoring high-risk patients
Source: Implement Sci. 2017 Jun 24;12:79. doi: 10.1186/s13012-017-0609-5 (PMC5483297; doi:10.1186/s13012-017-0609-5)
Supplement: Supplementary file 1 — Script for data collection on design seeds. (DOCX 19 kb) [file 13012_2017_609_MOESM1_ESM.docx]

**Script:**

Thank you for taking the time to meet with me today. We have the opportunity to improve patient monitoring in your clinic. In order to meet your needs for improved monitoring, we’d like to validate our findings and get your feedback.

Specifically, we would like to follow-up on the discussion you had with George Su regarding vulnerabilities of monitoring patients with high-risk conditions/ receiving high-risk treatment in your clinic. We’ve found that many other subspecialty clinics are facing similar challenges. .

We’d like to get your feedback on potential technology and organizational solutions that may alleviate the problems experienced in managing high-risk populations. Did we appropriately capture the problems you experience in your clinic? Do you think our suggested solutions will improve patient monitoring and efficiency?

As we go through the exercise, I’d like to remind you that there is no correct or incorrect answer. Your responses allow us to get a better sense of your experiences monitoring high-risk situations in clinic.

We expect this feedback collection exercise will take about 30 minutes. Thank you for your input!

**Instructions:**

*Part I*

First, we’ll look at a list of the problems we heard about from each clinic. **As you look at these problems, make a check next to those that you experience. You can circle problems that are even more relevant and make notes on these cards.**

*Part II*

We’ll now look at a set of cards that list 13 suggestions to improve patient monitoring. The solutions respond to issues raised by at least one of the clinics we visited.

**From your vantage point at your clinic, think about patients who are at high-risk of being lost to follow-up and/ or require multiple steps or high effort to track/ monitor.**

**We’ll also ask you to prioritize the cards according to importance. If you think a solution characteristic is particularly important, you can mark it with an asterisk so it is easier to rank them at the end of the exercise. We also encourage you to take notes or talk aloud if any other ideas come to mind.** This will allow us to both refine our findings and better understand the differences between clinics.

Here is the first solution card. All 13 look like this. This *(pointing)* is a statement from a specialty clinic’s viewpoint that motivates the solution attribute. The summarized solution attribute is in bold *(pointing)*, followed by details related to the solution attribute *(pointing*). After you read the quoted statement and information in each box, please respond to the statements at the bottom. You can do this after I tell you one more thing. As you go through all the cards, please order them so that you end up with the most important solution card on the top and least important one on the bottom. It is fine to move them around as you go, or to go back through at the end. I will mark the cards 1-13 according to your final order. If we have time after the exercise, I’ll ask you to explain why you ranked in the order that you did.

*Ask for:*

Additional comments – reasons for ratings, choices, and thoughts about any of the potential solutions

What clinic is participant representing?
