# Supplementary material for: Implementation science for ambulatory care safety: a novel method to develop context-sensitive interventions to reduce quality gaps in monitoring high-risk patients
Source: Implement Sci. 2017 Jun 24;12:79. doi: 10.1186/s13012-017-0609-5 (PMC5483297; doi:10.1186/s13012-017-0609-5)
Supplement: Supplementary file 2 — Feedback form for data collection on design seeds. (PPTX 135 kb) [file 13012_2017_609_MOESM2_ESM.pptx]

## Slide 1
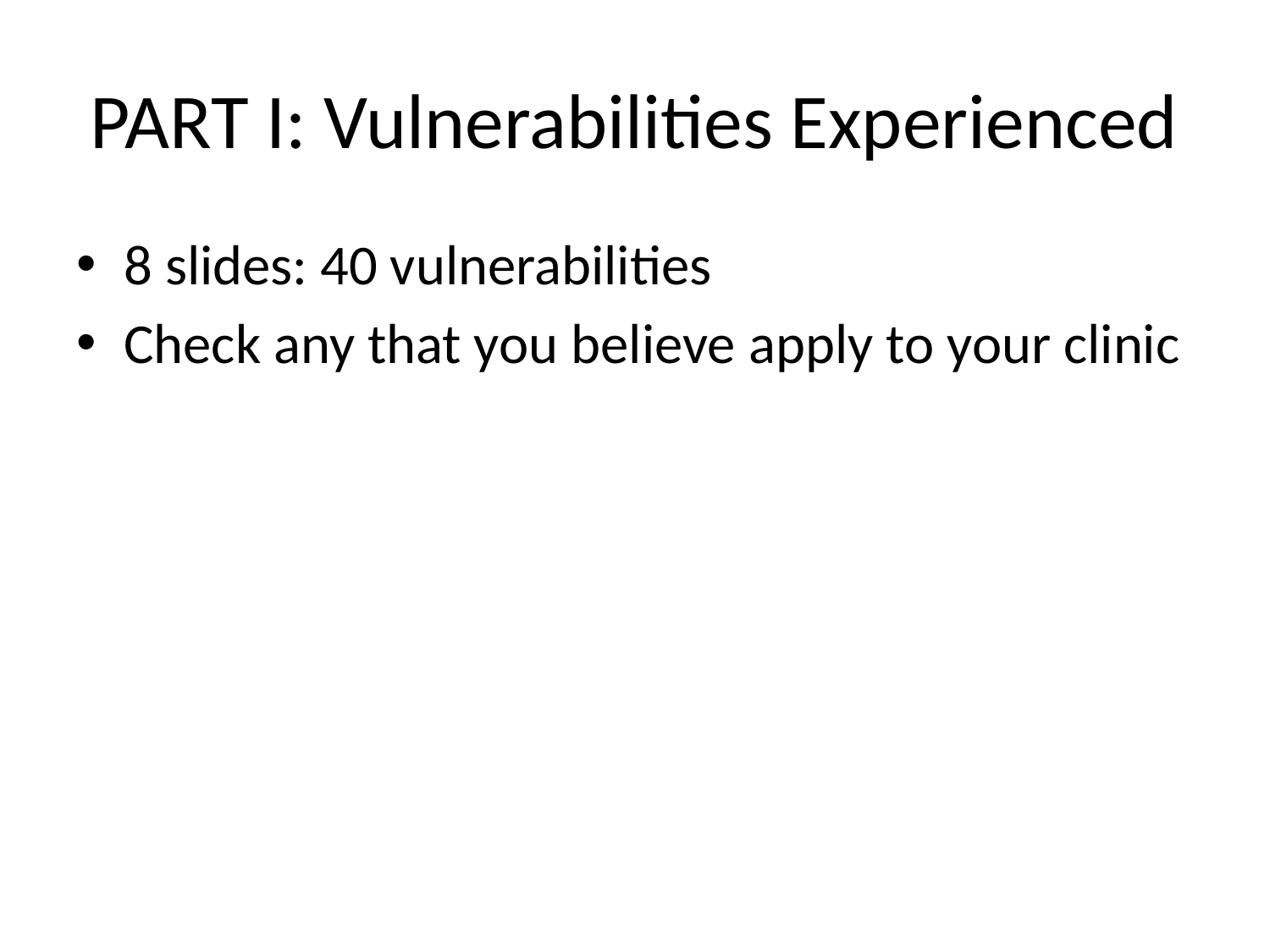

# PART I: Vulnerabilities Experienced
8 slides: 40 vulnerabilities
Check any that you believe apply to your clinic

## Slide 2
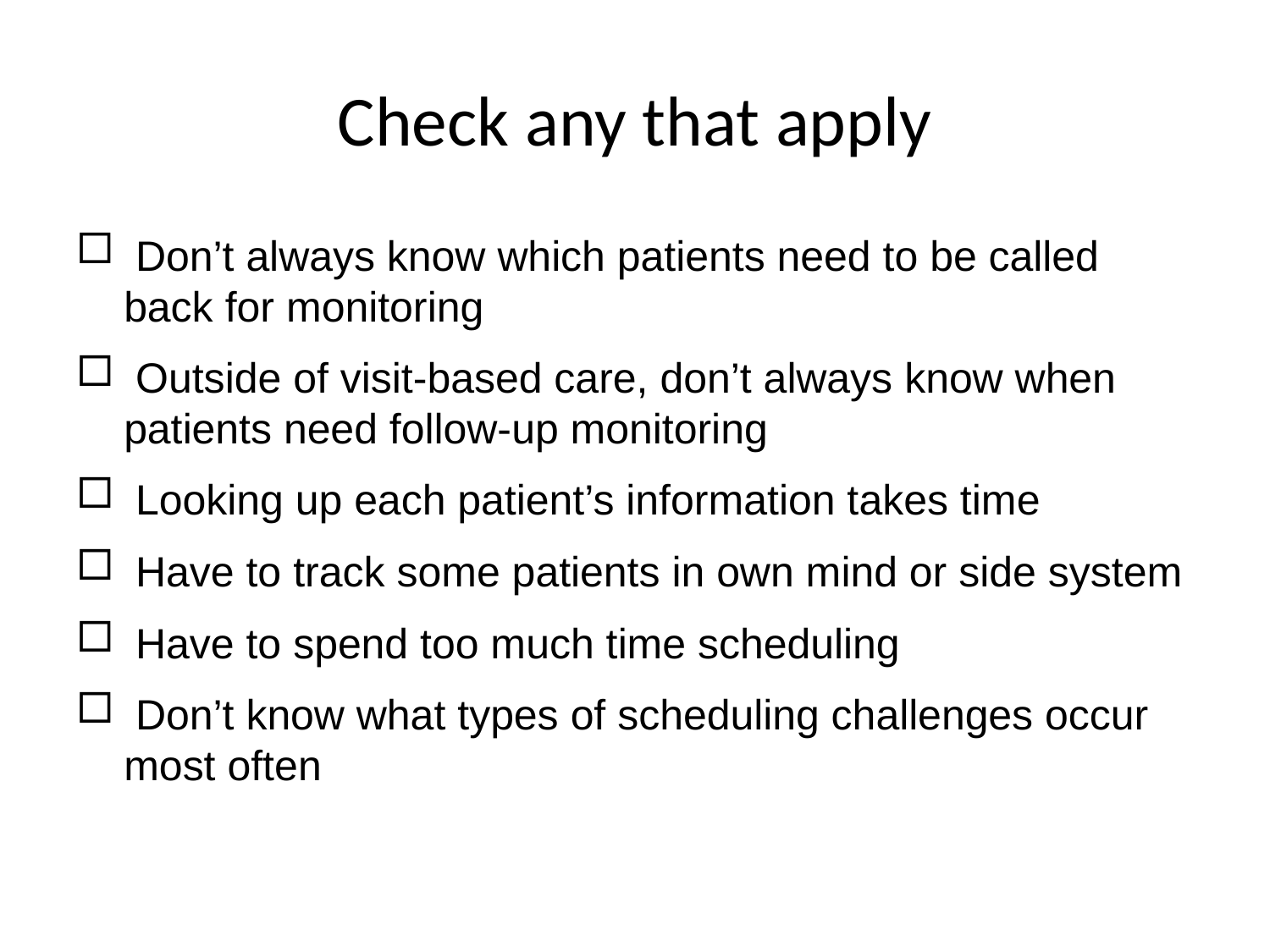

# Check any that apply
 Don’t always know which patients need to be called back for monitoring
 Outside of visit-based care, don’t always know when patients need follow-up monitoring
 Looking up each patient’s information takes time
 Have to track some patients in own mind or side system
 Have to spend too much time scheduling
 Don’t know what types of scheduling challenges occur most often

## Slide 3
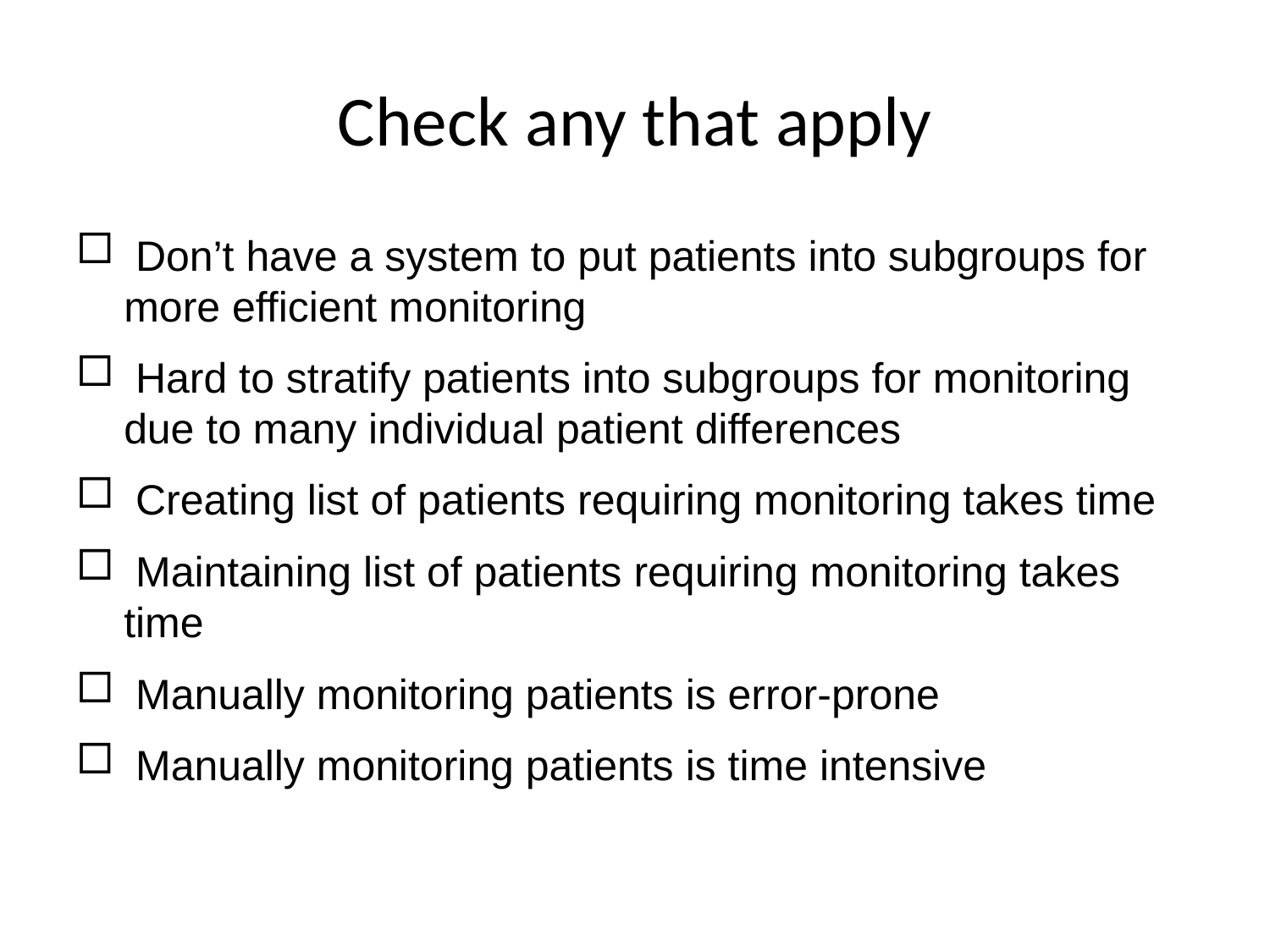

# Check any that apply
 Don’t have a system to put patients into subgroups for more efficient monitoring
 Hard to stratify patients into subgroups for monitoring due to many individual patient differences
 Creating list of patients requiring monitoring takes time
 Maintaining list of patients requiring monitoring takes time
 Manually monitoring patients is error-prone
 Manually monitoring patients is time intensive

## Slide 4
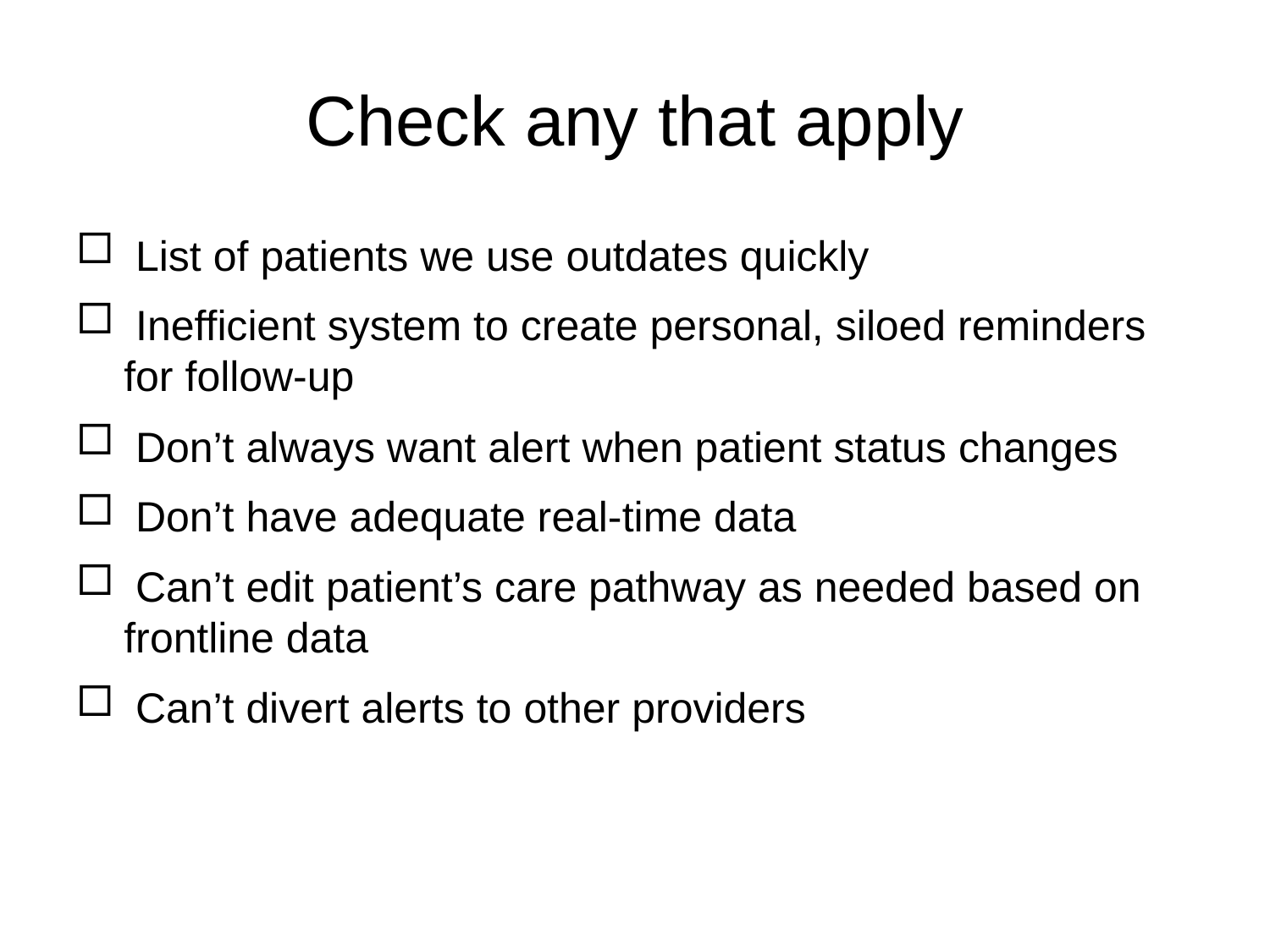

# Check any that apply
 List of patients we use outdates quickly
 Inefficient system to create personal, siloed reminders for follow-up
 Don’t always want alert when patient status changes
 Don’t have adequate real-time data
 Can’t edit patient’s care pathway as needed based on frontline data
 Can’t divert alerts to other providers

## Slide 5
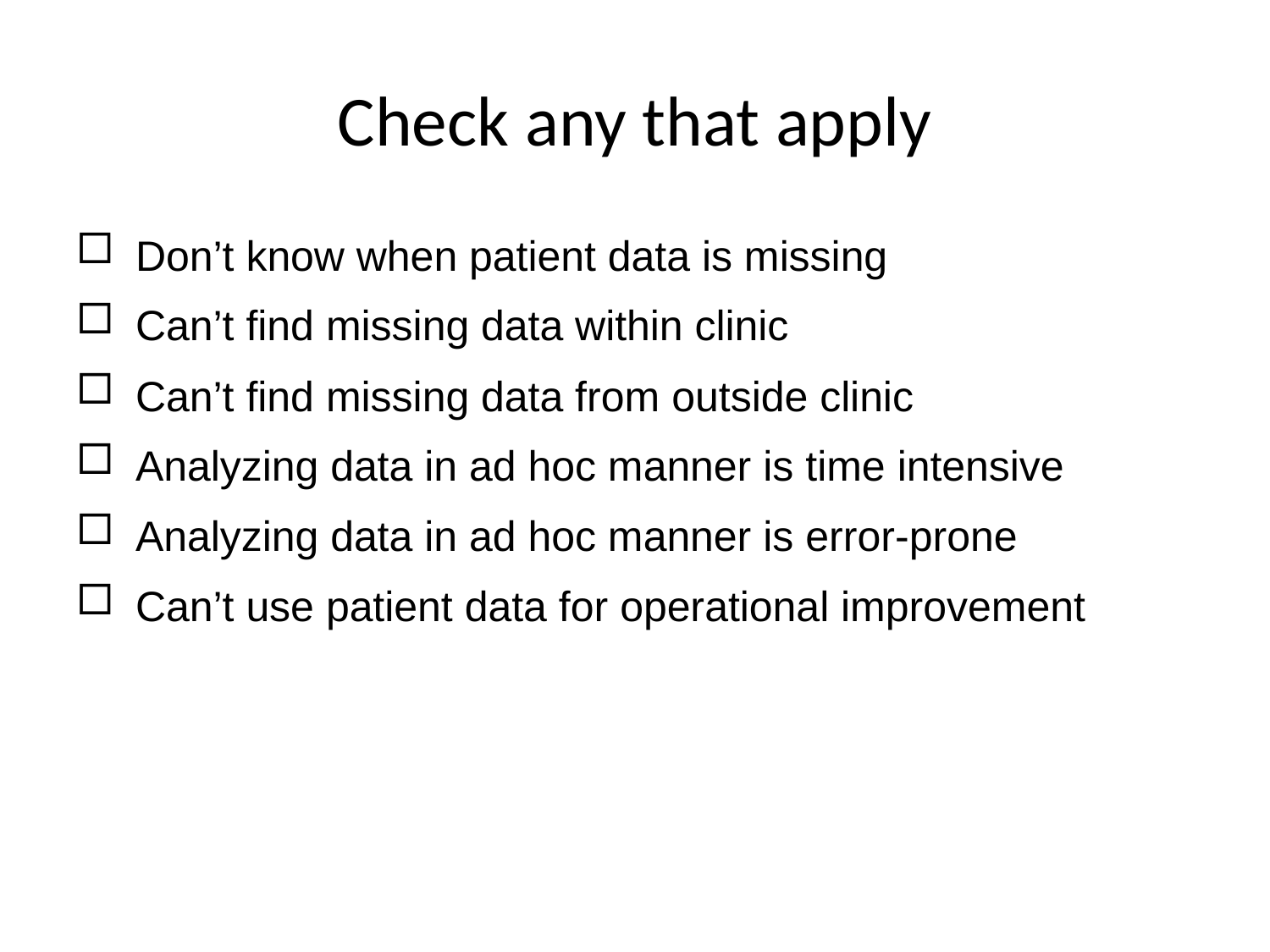

# Check any that apply
 Don’t know when patient data is missing
 Can’t find missing data within clinic
 Can’t find missing data from outside clinic
 Analyzing data in ad hoc manner is time intensive
 Analyzing data in ad hoc manner is error-prone
 Can’t use patient data for operational improvement

## Slide 6
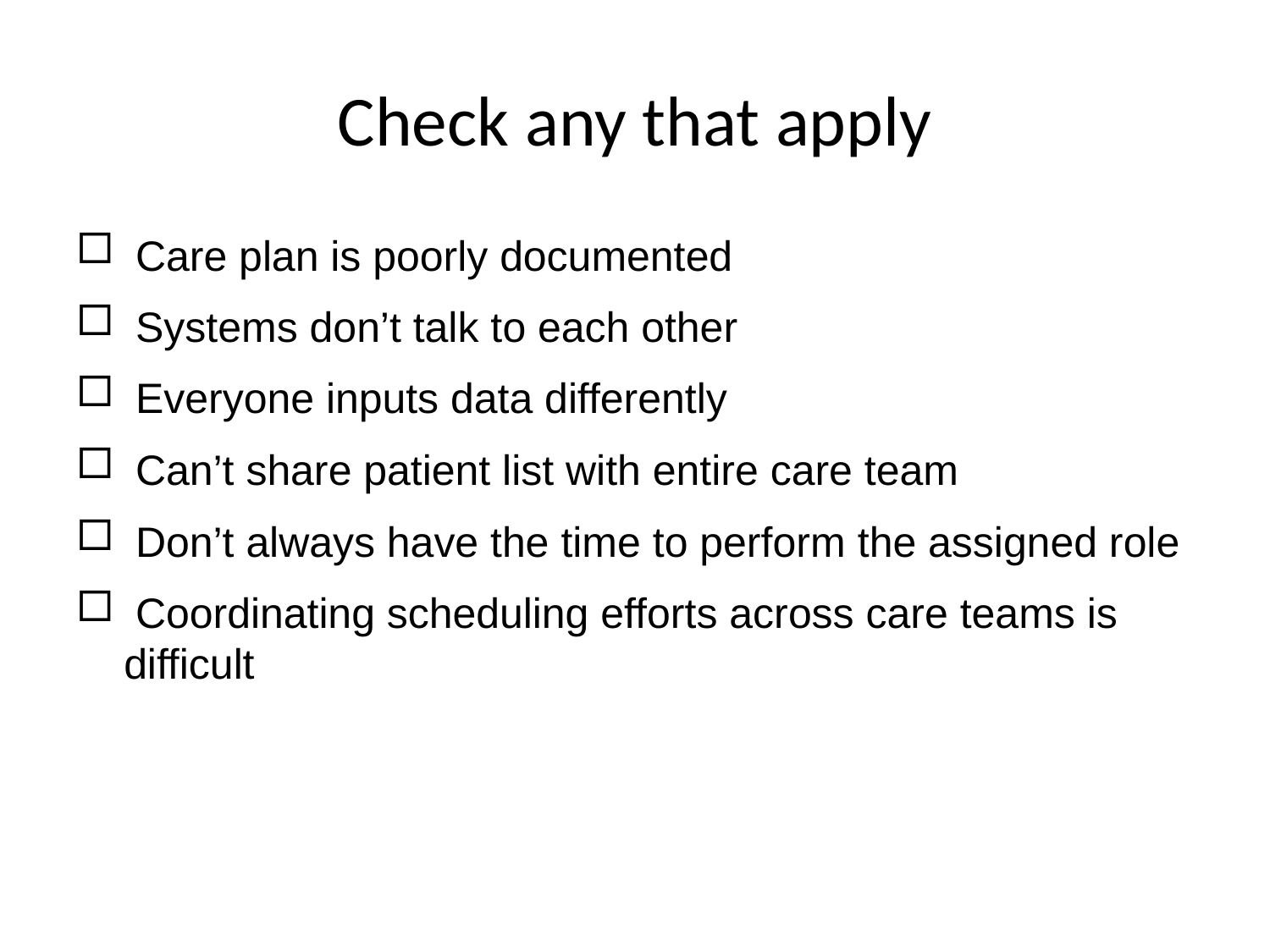

# Check any that apply
 Care plan is poorly documented
 Systems don’t talk to each other
 Everyone inputs data differently
 Can’t share patient list with entire care team
 Don’t always have the time to perform the assigned role
 Coordinating scheduling efforts across care teams is difficult

## Slide 7
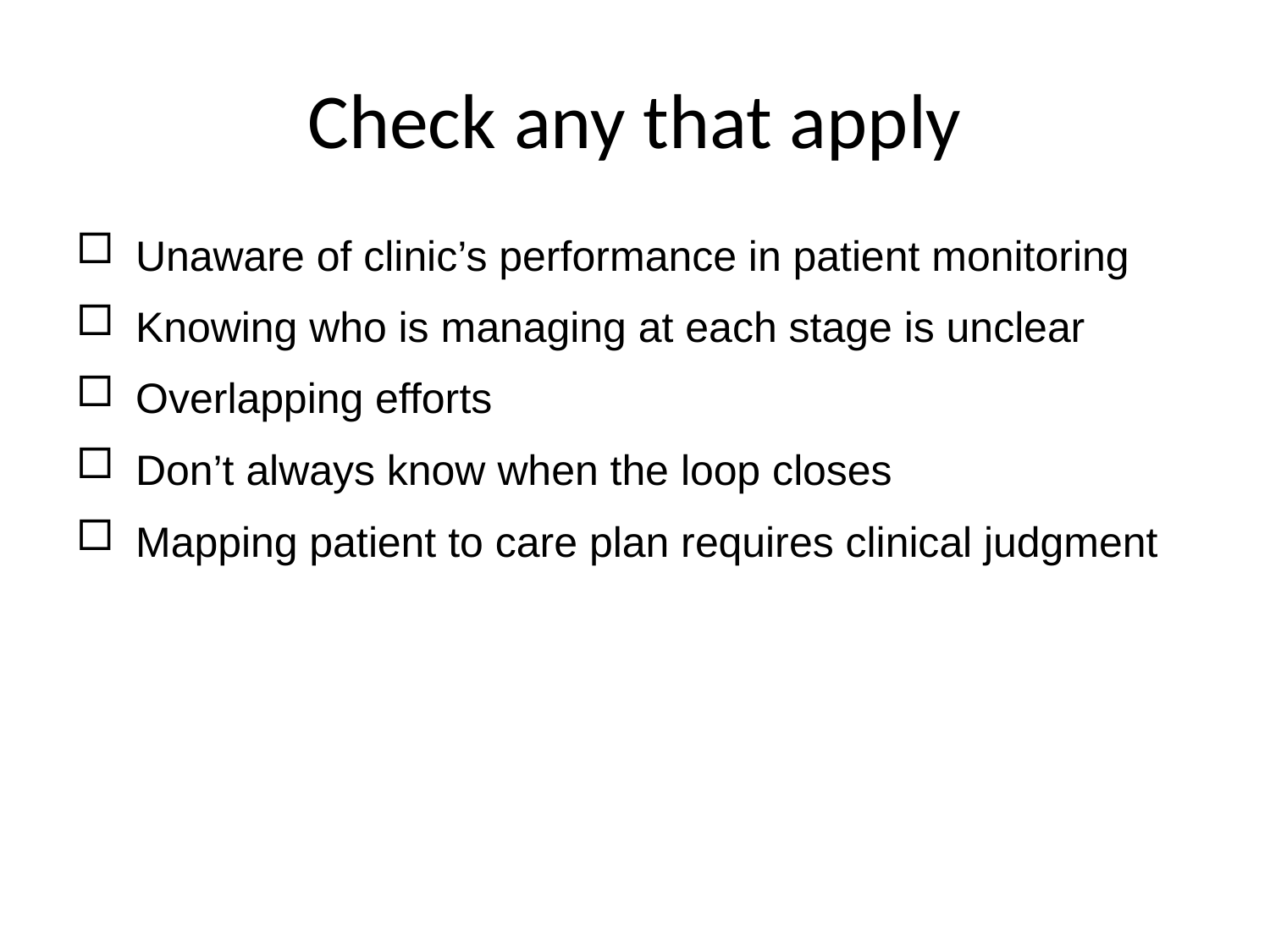

# Check any that apply
 Unaware of clinic’s performance in patient monitoring
 Knowing who is managing at each stage is unclear
 Overlapping efforts
 Don’t always know when the loop closes
 Mapping patient to care plan requires clinical judgment

## Slide 8
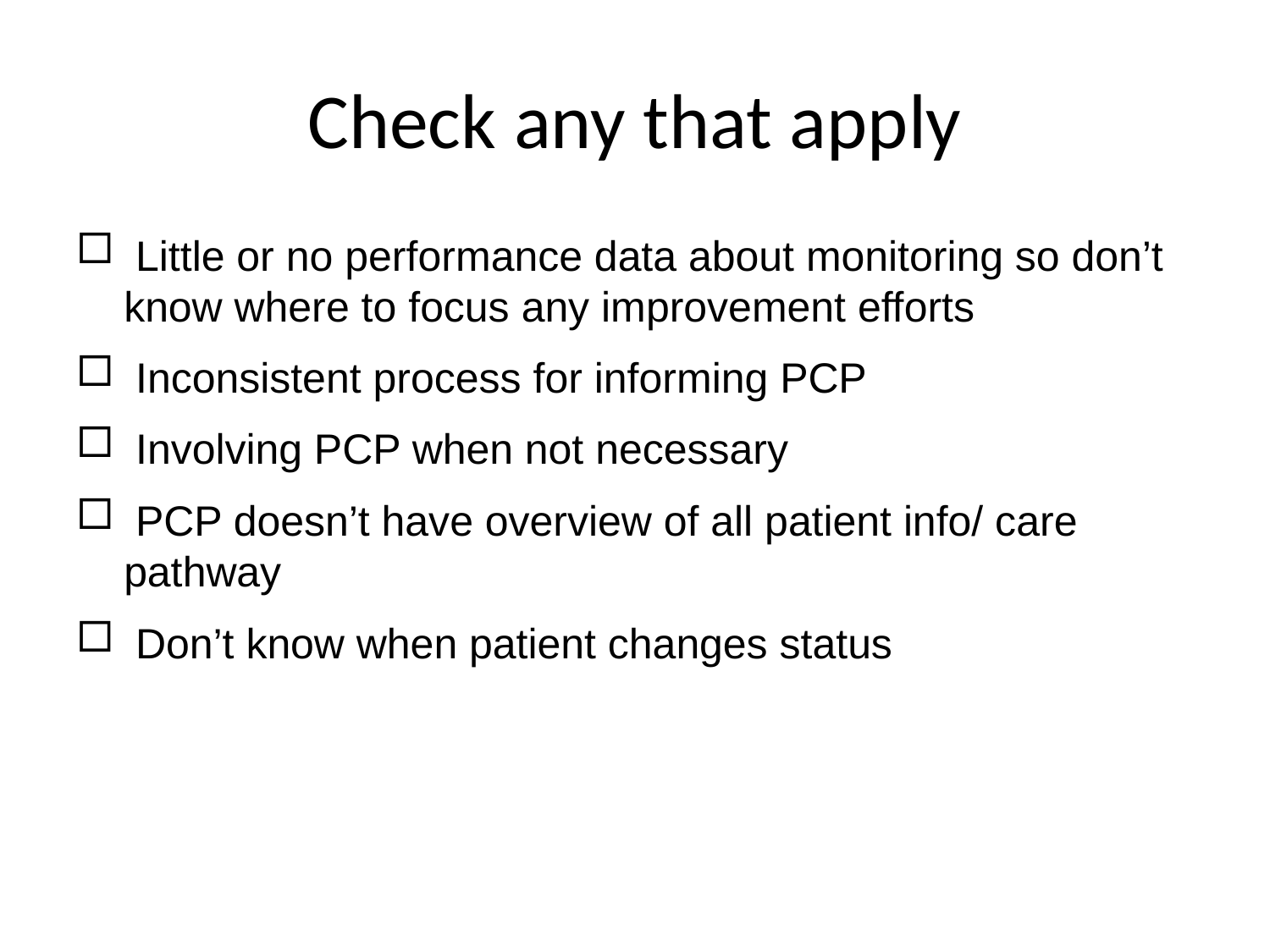

# Check any that apply
 Little or no performance data about monitoring so don’t know where to focus any improvement efforts
 Inconsistent process for informing PCP
 Involving PCP when not necessary
 PCP doesn’t have overview of all patient info/ care pathway
 Don’t know when patient changes status

## Slide 9
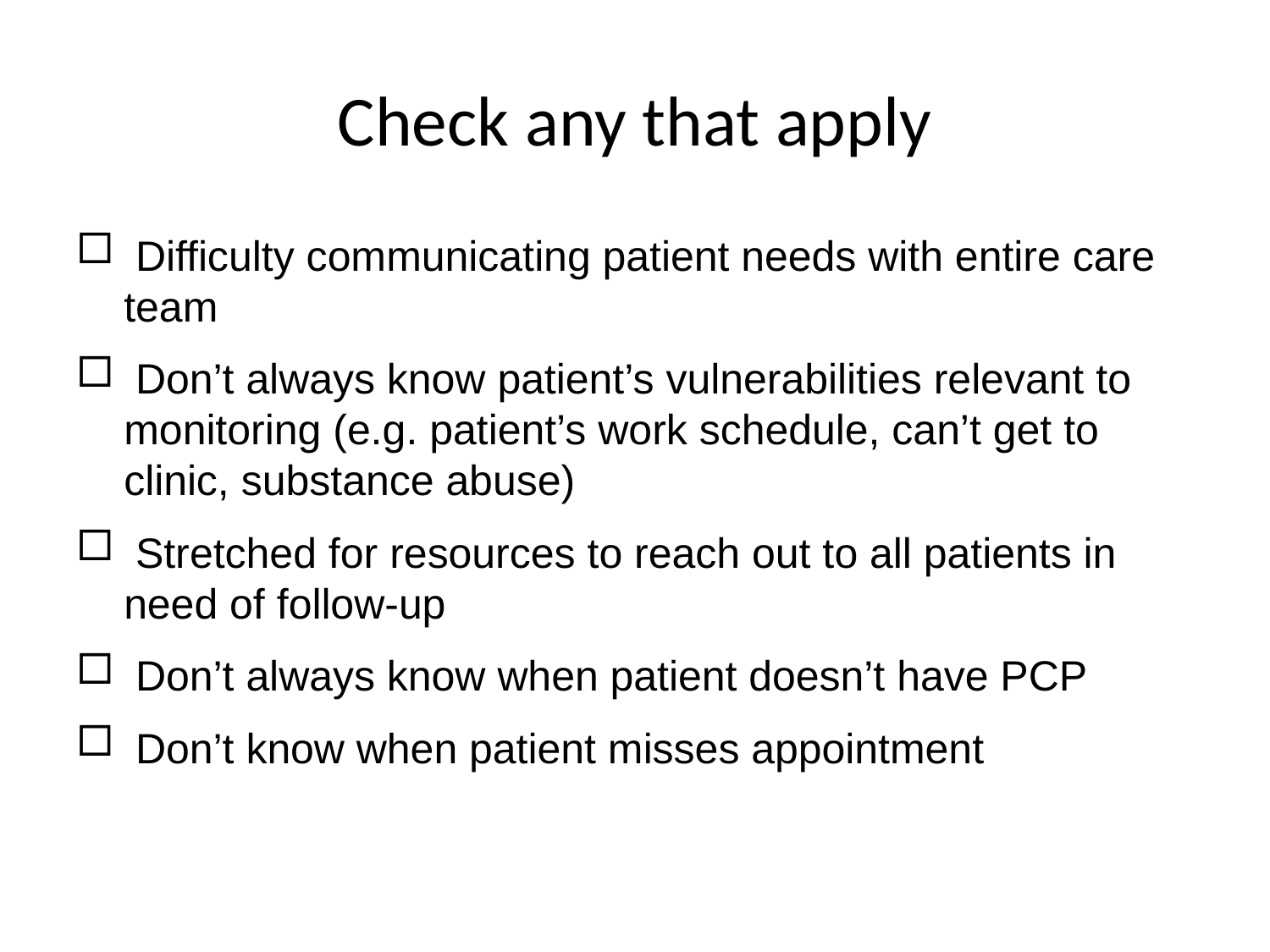

# Check any that apply
 Difficulty communicating patient needs with entire care team
 Don’t always know patient’s vulnerabilities relevant to monitoring (e.g. patient’s work schedule, can’t get to clinic, substance abuse)
 Stretched for resources to reach out to all patients in need of follow-up
 Don’t always know when patient doesn’t have PCP
 Don’t know when patient misses appointment

## Slide 10
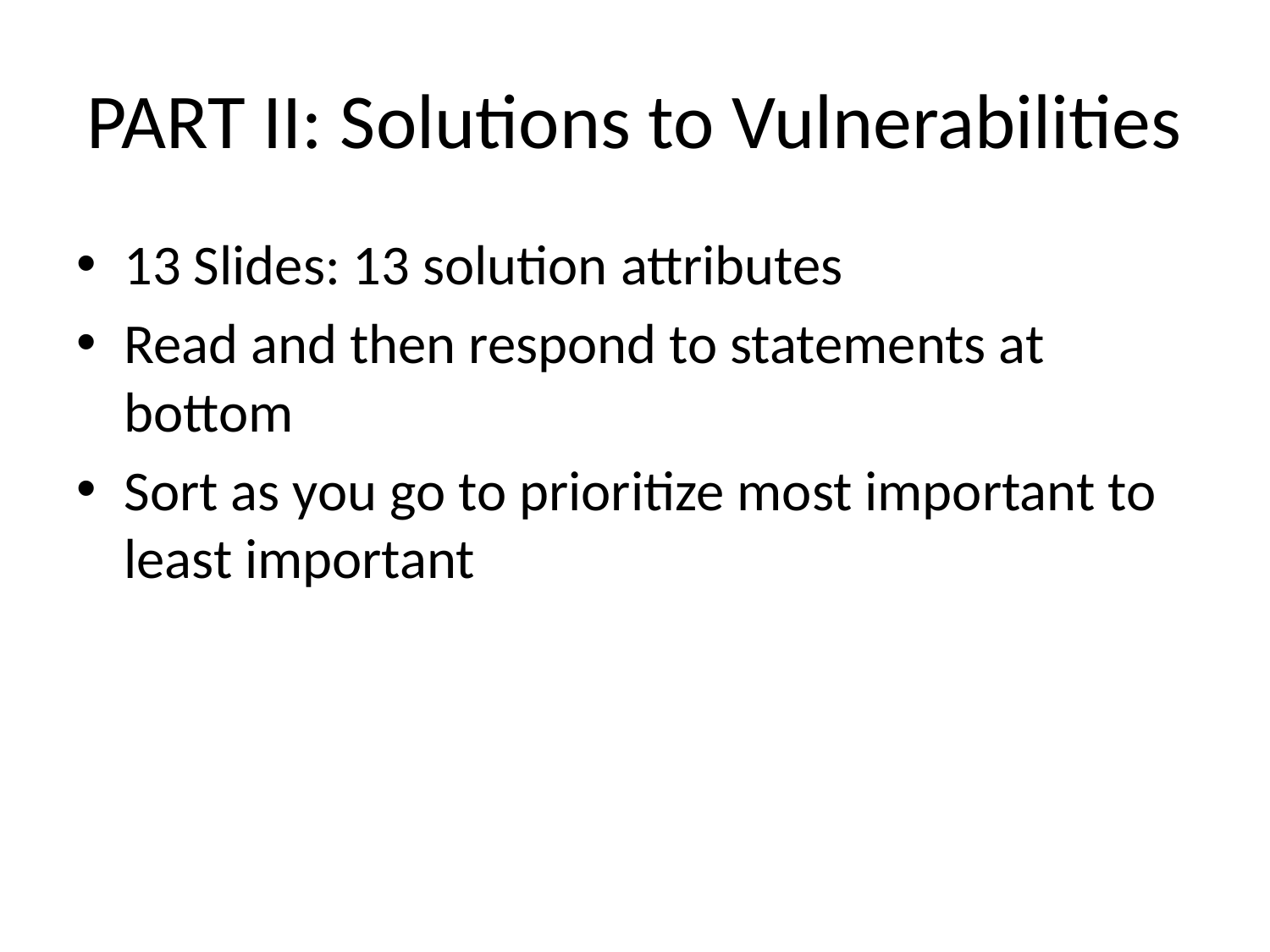

# PART II: Solutions to Vulnerabilities
13 Slides: 13 solution attributes
Read and then respond to statements at bottom
Sort as you go to prioritize most important to least important

## Slide 11
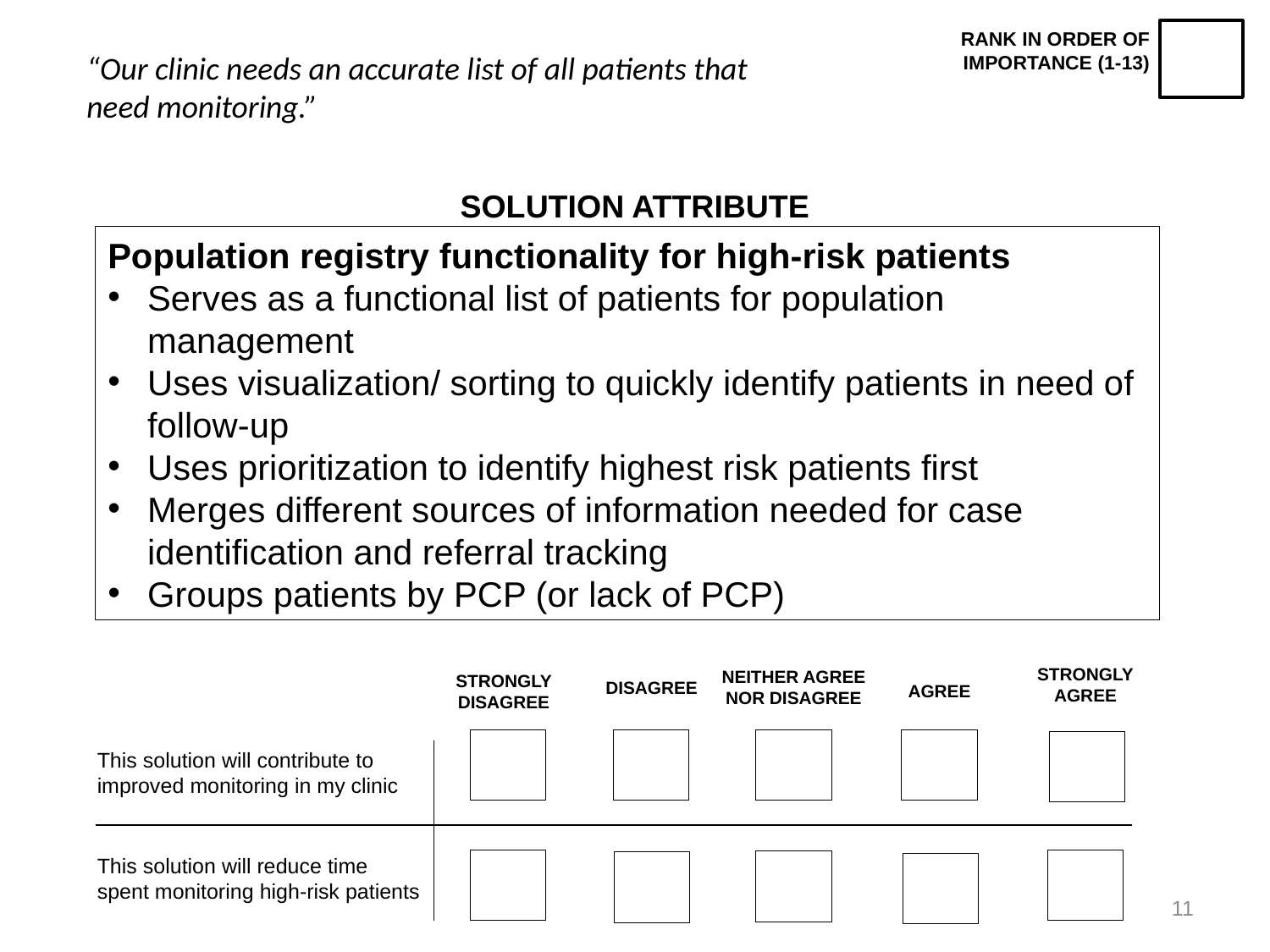

RANK IN ORDER OF IMPORTANCE (1-13)
“Our clinic needs an accurate list of all patients that need monitoring.”
SOLUTION ATTRIBUTE
Population registry functionality for high-risk patients
Serves as a functional list of patients for population management
Uses visualization/ sorting to quickly identify patients in need of follow-up
Uses prioritization to identify highest risk patients first
Merges different sources of information needed for case identification and referral tracking
Groups patients by PCP (or lack of PCP)
STRONGLY AGREE
NEITHER AGREE NOR DISAGREE
STRONGLY DISAGREE
DISAGREE
AGREE
This solution will contribute to improved monitoring in my clinic
This solution will reduce time spent monitoring high-risk patients
11

## Slide 12
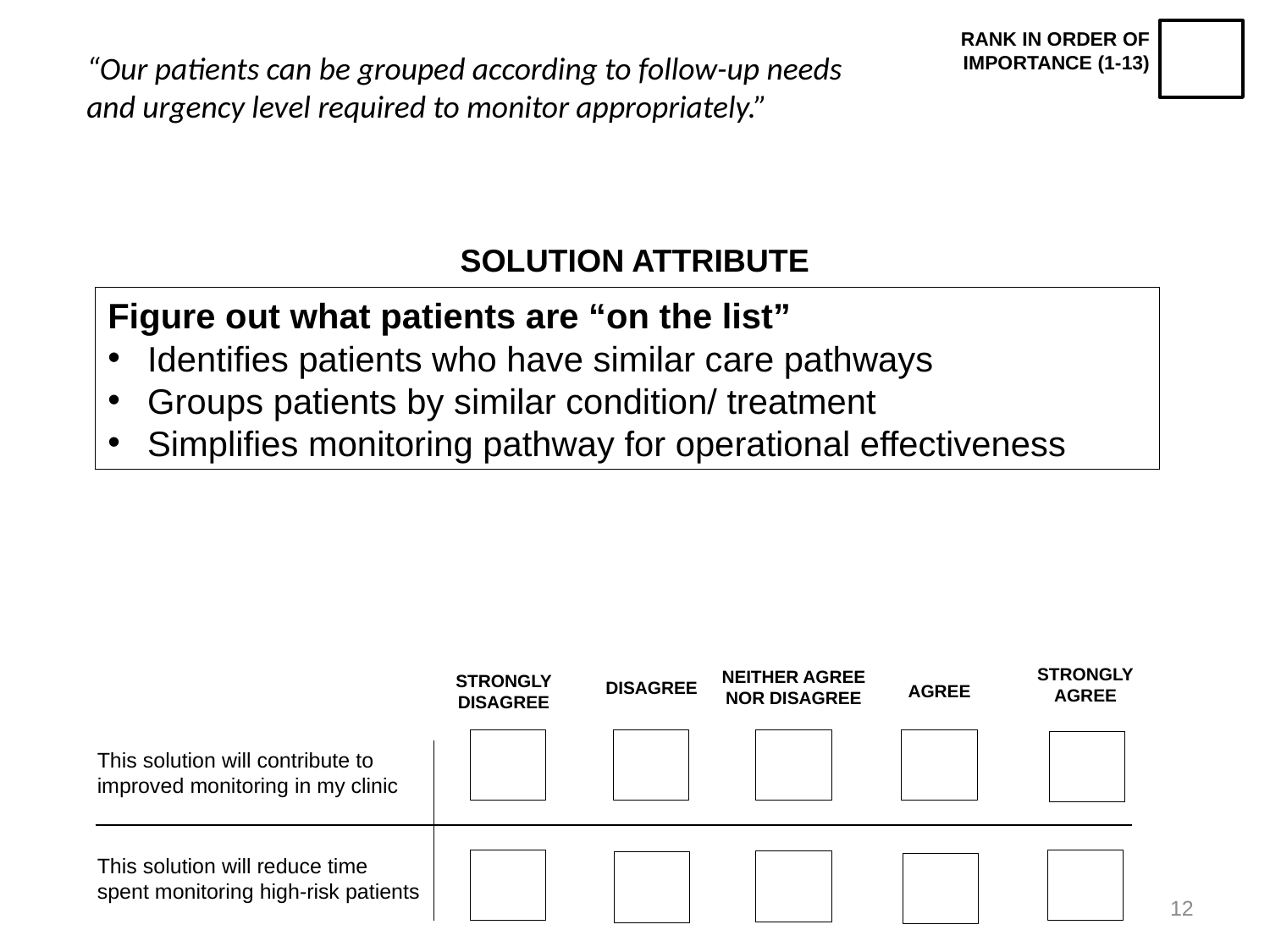

RANK IN ORDER OF IMPORTANCE (1-13)
“Our patients can be grouped according to follow-up needs and urgency level required to monitor appropriately.”
SOLUTION ATTRIBUTE
Figure out what patients are “on the list”
Identifies patients who have similar care pathways
Groups patients by similar condition/ treatment
Simplifies monitoring pathway for operational effectiveness
STRONGLY AGREE
NEITHER AGREE NOR DISAGREE
STRONGLY DISAGREE
DISAGREE
AGREE
This solution will contribute to improved monitoring in my clinic
This solution will reduce time spent monitoring high-risk patients
12

## Slide 13
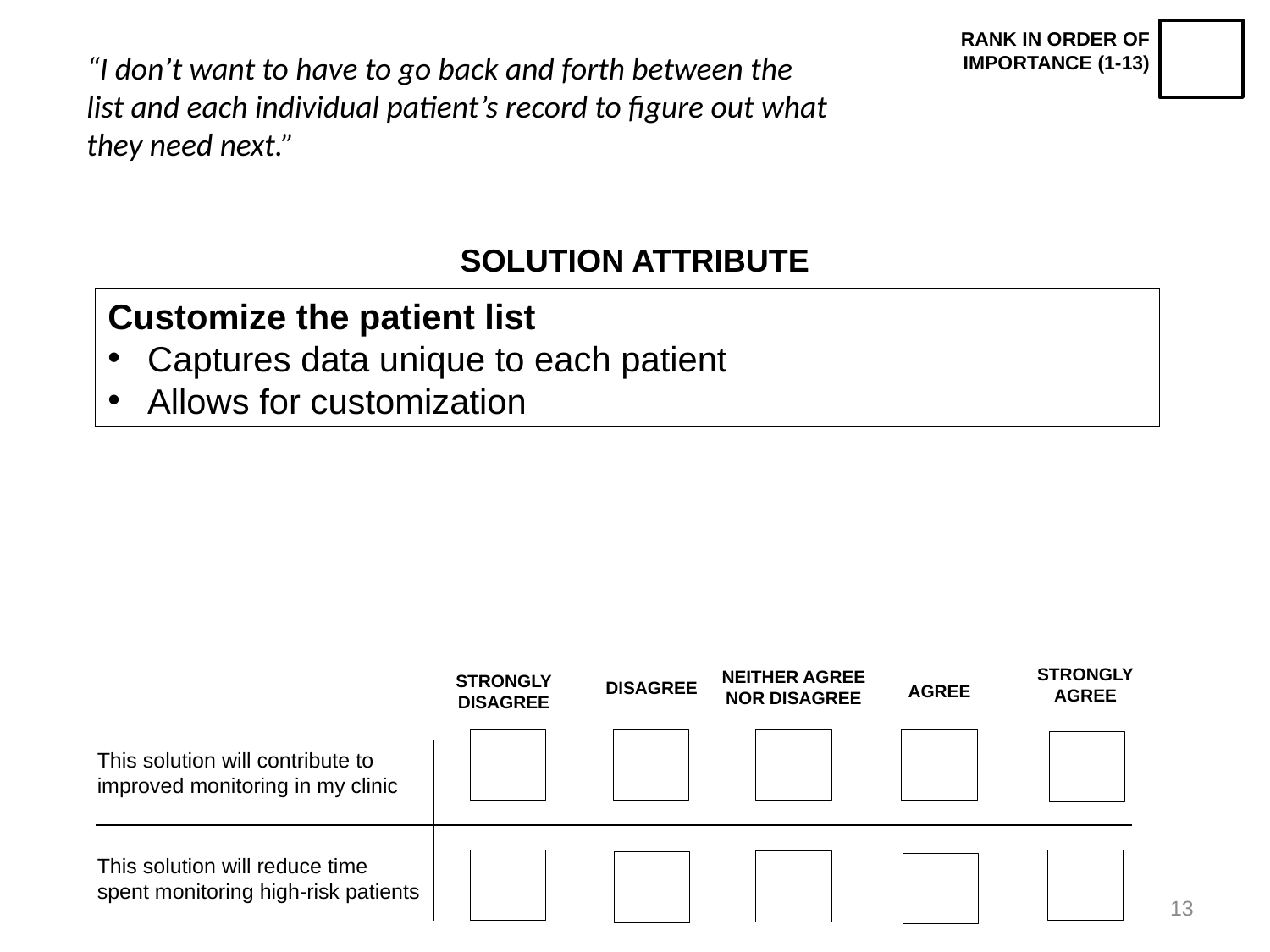

RANK IN ORDER OF IMPORTANCE (1-13)
“I don’t want to have to go back and forth between the list and each individual patient’s record to figure out what they need next.”
SOLUTION ATTRIBUTE
Customize the patient list
Captures data unique to each patient
Allows for customization
STRONGLY AGREE
NEITHER AGREE NOR DISAGREE
STRONGLY DISAGREE
DISAGREE
AGREE
This solution will contribute to improved monitoring in my clinic
This solution will reduce time spent monitoring high-risk patients
13

## Slide 14
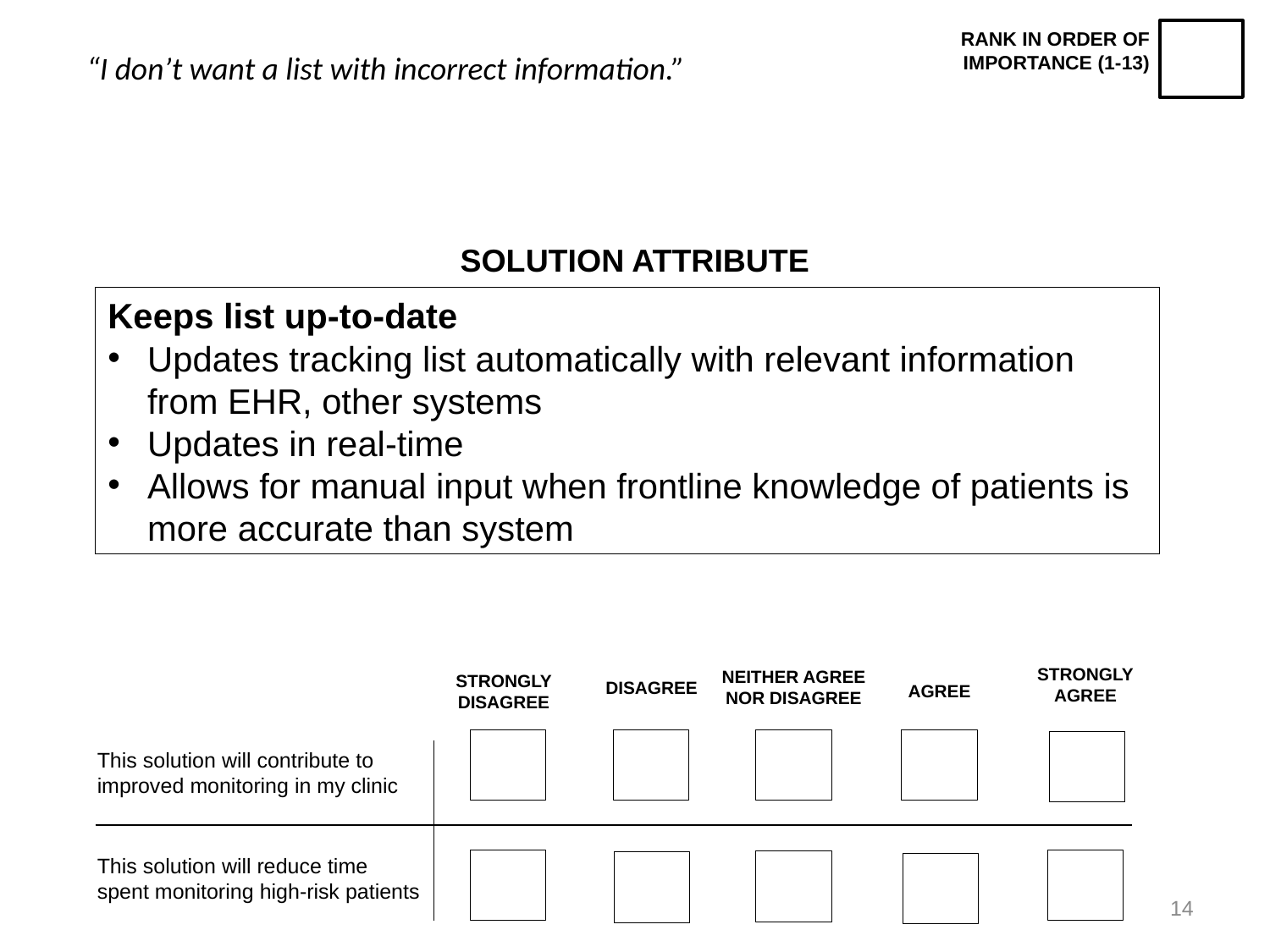

RANK IN ORDER OF IMPORTANCE (1-13)
“I don’t want a list with incorrect information.”
SOLUTION ATTRIBUTE
Keeps list up-to-date
Updates tracking list automatically with relevant information from EHR, other systems
Updates in real-time
Allows for manual input when frontline knowledge of patients is more accurate than system
STRONGLY AGREE
NEITHER AGREE NOR DISAGREE
STRONGLY DISAGREE
DISAGREE
AGREE
This solution will contribute to improved monitoring in my clinic
This solution will reduce time spent monitoring high-risk patients
14

## Slide 15
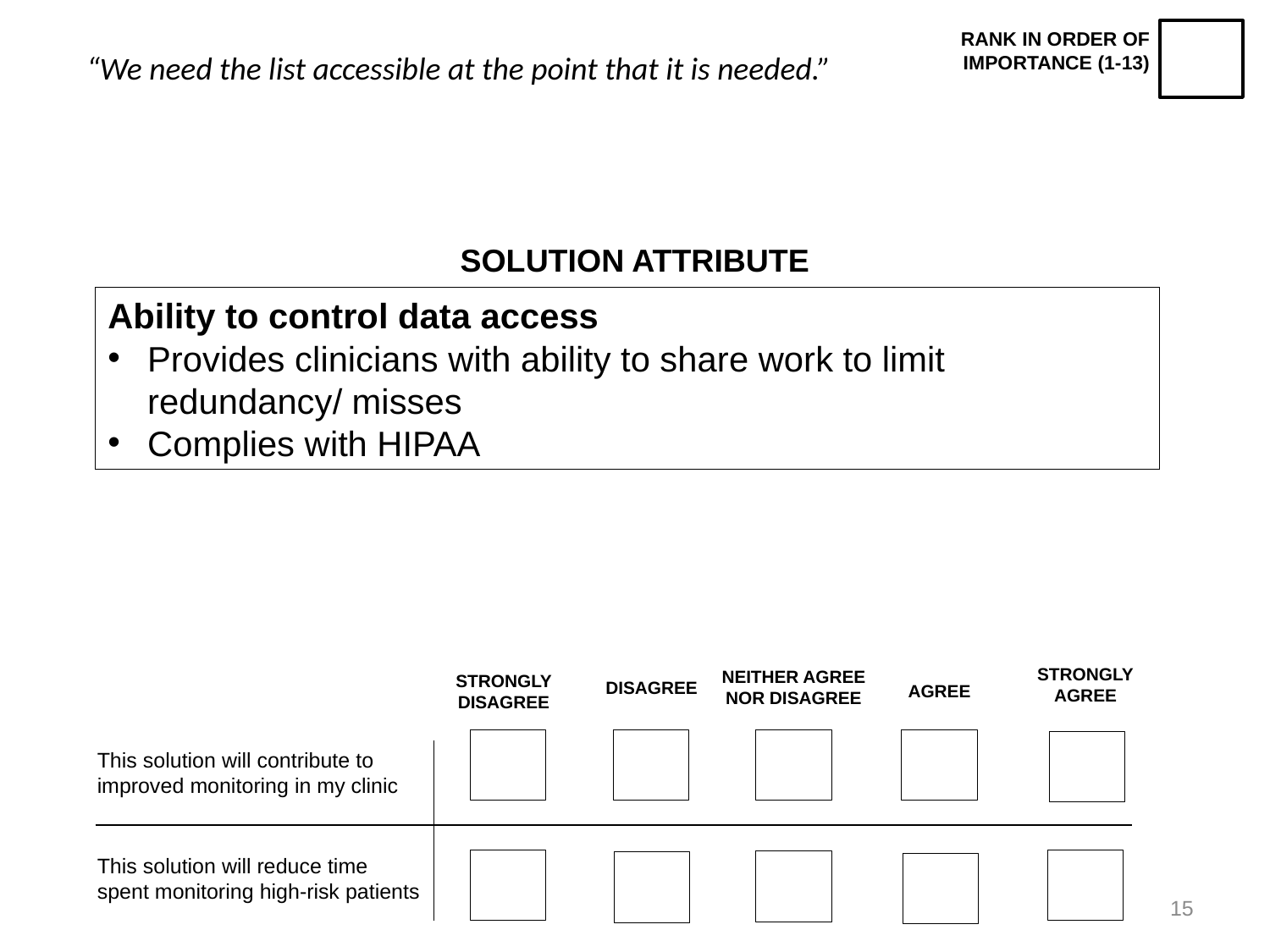

RANK IN ORDER OF IMPORTANCE (1-13)
“We need the list accessible at the point that it is needed.”
SOLUTION ATTRIBUTE
Ability to control data access
Provides clinicians with ability to share work to limit redundancy/ misses
Complies with HIPAA
STRONGLY AGREE
NEITHER AGREE NOR DISAGREE
STRONGLY DISAGREE
DISAGREE
AGREE
This solution will contribute to improved monitoring in my clinic
This solution will reduce time spent monitoring high-risk patients
15

## Slide 16
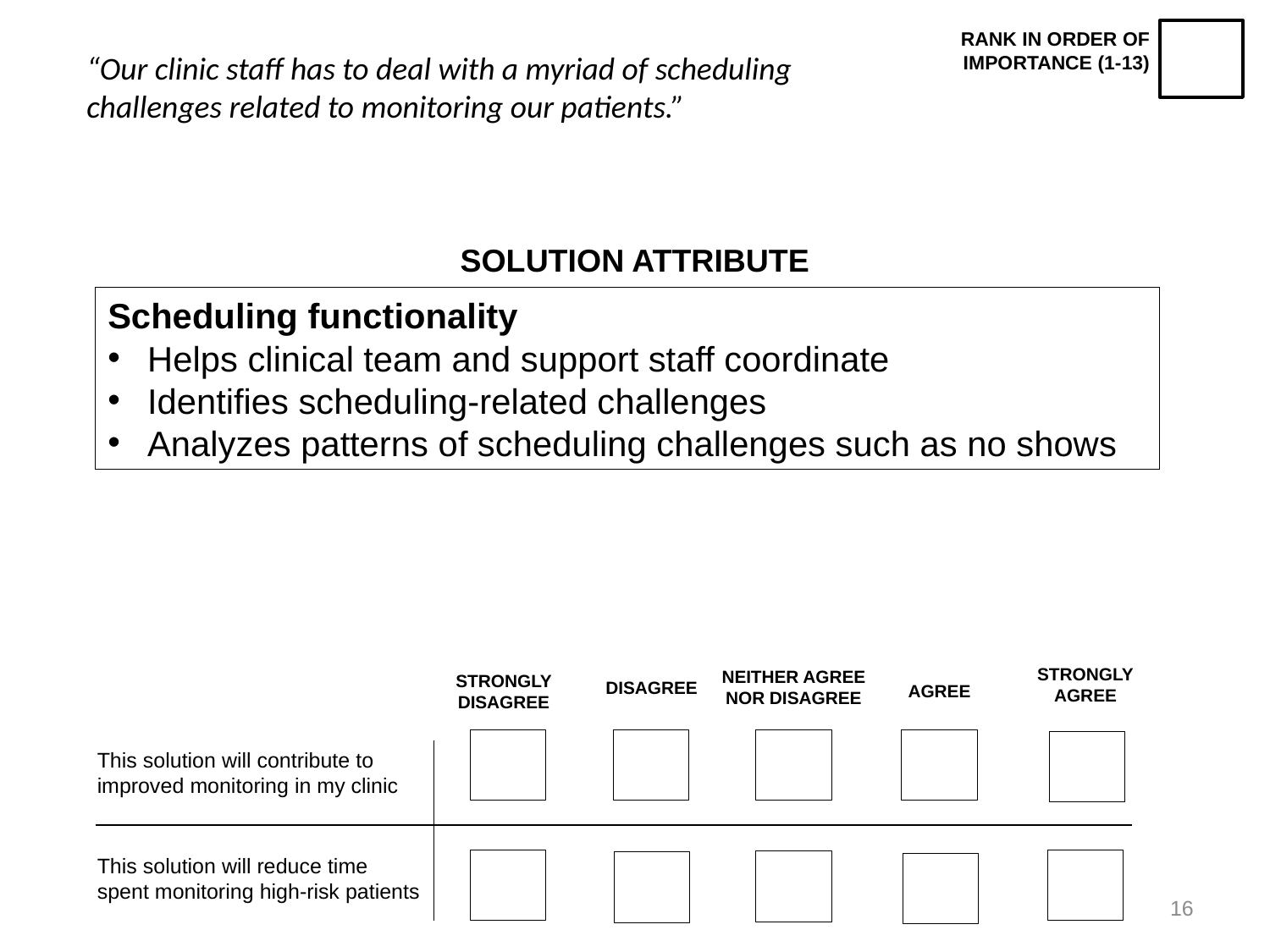

RANK IN ORDER OF IMPORTANCE (1-13)
“Our clinic staff has to deal with a myriad of scheduling challenges related to monitoring our patients.”
SOLUTION ATTRIBUTE
Scheduling functionality
Helps clinical team and support staff coordinate
Identifies scheduling-related challenges
Analyzes patterns of scheduling challenges such as no shows
STRONGLY AGREE
NEITHER AGREE NOR DISAGREE
STRONGLY DISAGREE
DISAGREE
AGREE
This solution will contribute to improved monitoring in my clinic
This solution will reduce time spent monitoring high-risk patients
16

## Slide 17
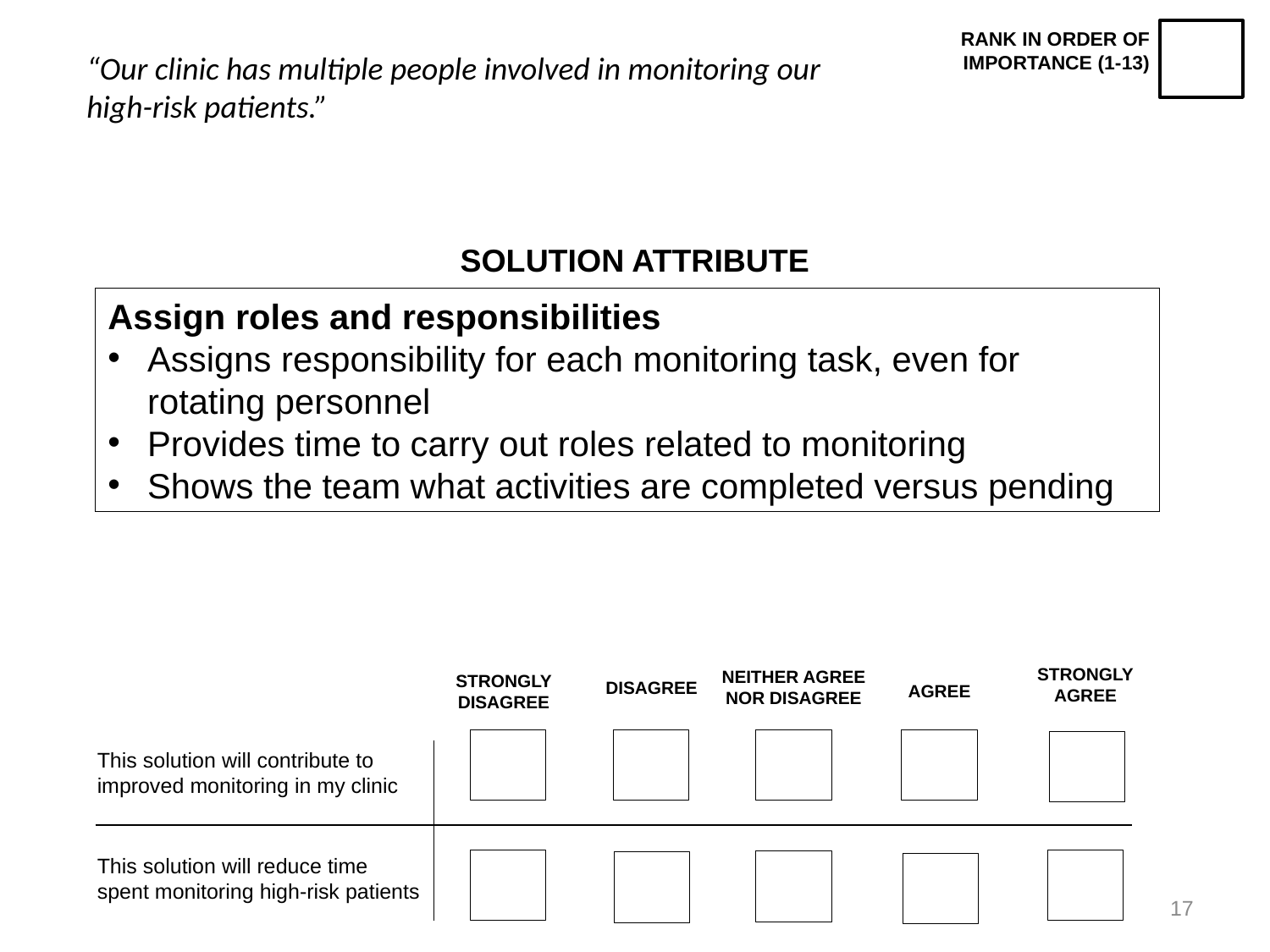

RANK IN ORDER OF IMPORTANCE (1-13)
“Our clinic has multiple people involved in monitoring our high-risk patients.”
SOLUTION ATTRIBUTE
Assign roles and responsibilities
Assigns responsibility for each monitoring task, even for rotating personnel
Provides time to carry out roles related to monitoring
Shows the team what activities are completed versus pending
STRONGLY AGREE
NEITHER AGREE NOR DISAGREE
STRONGLY DISAGREE
DISAGREE
AGREE
This solution will contribute to improved monitoring in my clinic
This solution will reduce time spent monitoring high-risk patients
17

## Slide 18
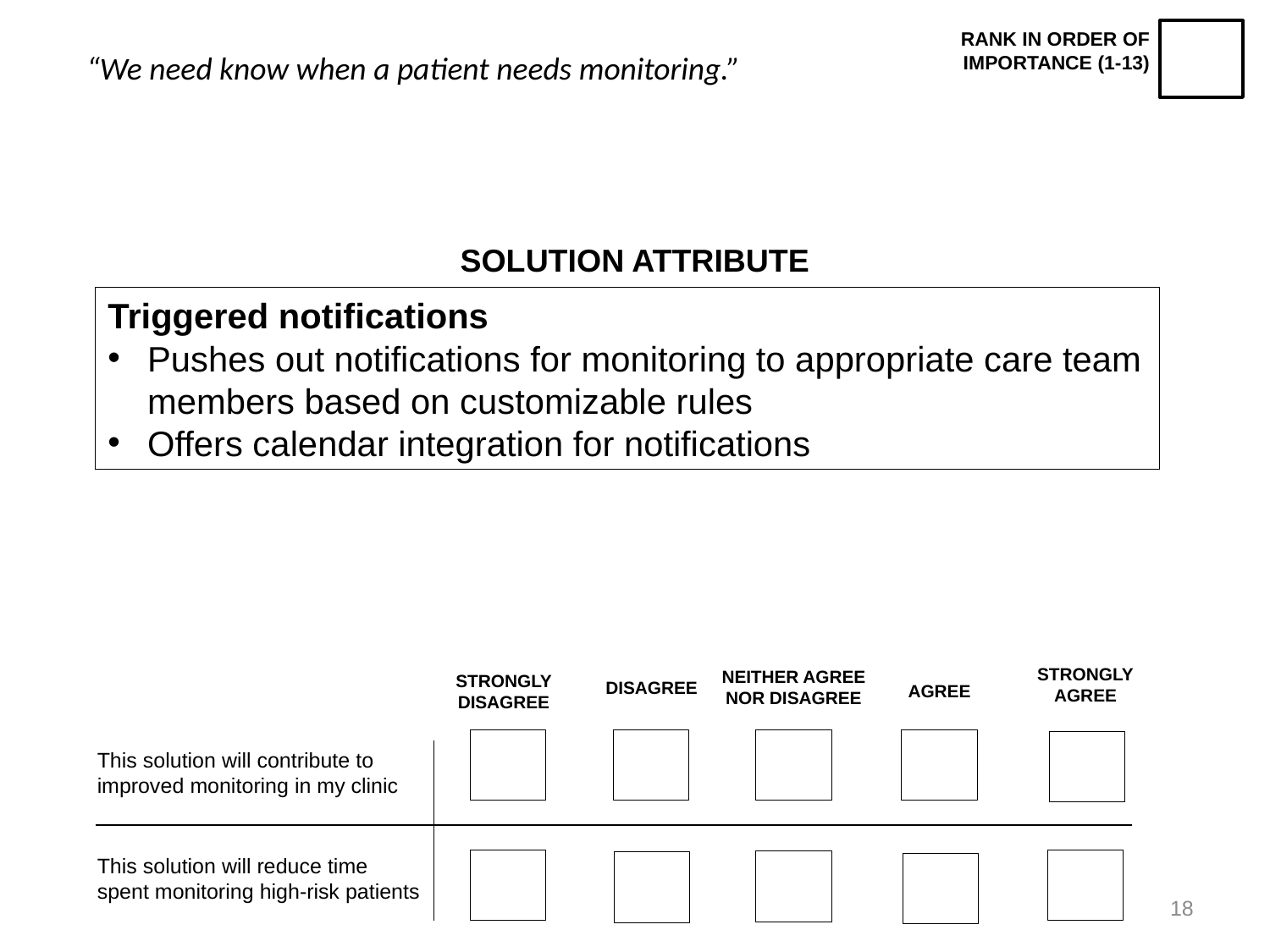

RANK IN ORDER OF IMPORTANCE (1-13)
“We need know when a patient needs monitoring.”
SOLUTION ATTRIBUTE
Triggered notifications
Pushes out notifications for monitoring to appropriate care team members based on customizable rules
Offers calendar integration for notifications
STRONGLY AGREE
NEITHER AGREE NOR DISAGREE
STRONGLY DISAGREE
DISAGREE
AGREE
This solution will contribute to improved monitoring in my clinic
This solution will reduce time spent monitoring high-risk patients
18

## Slide 19
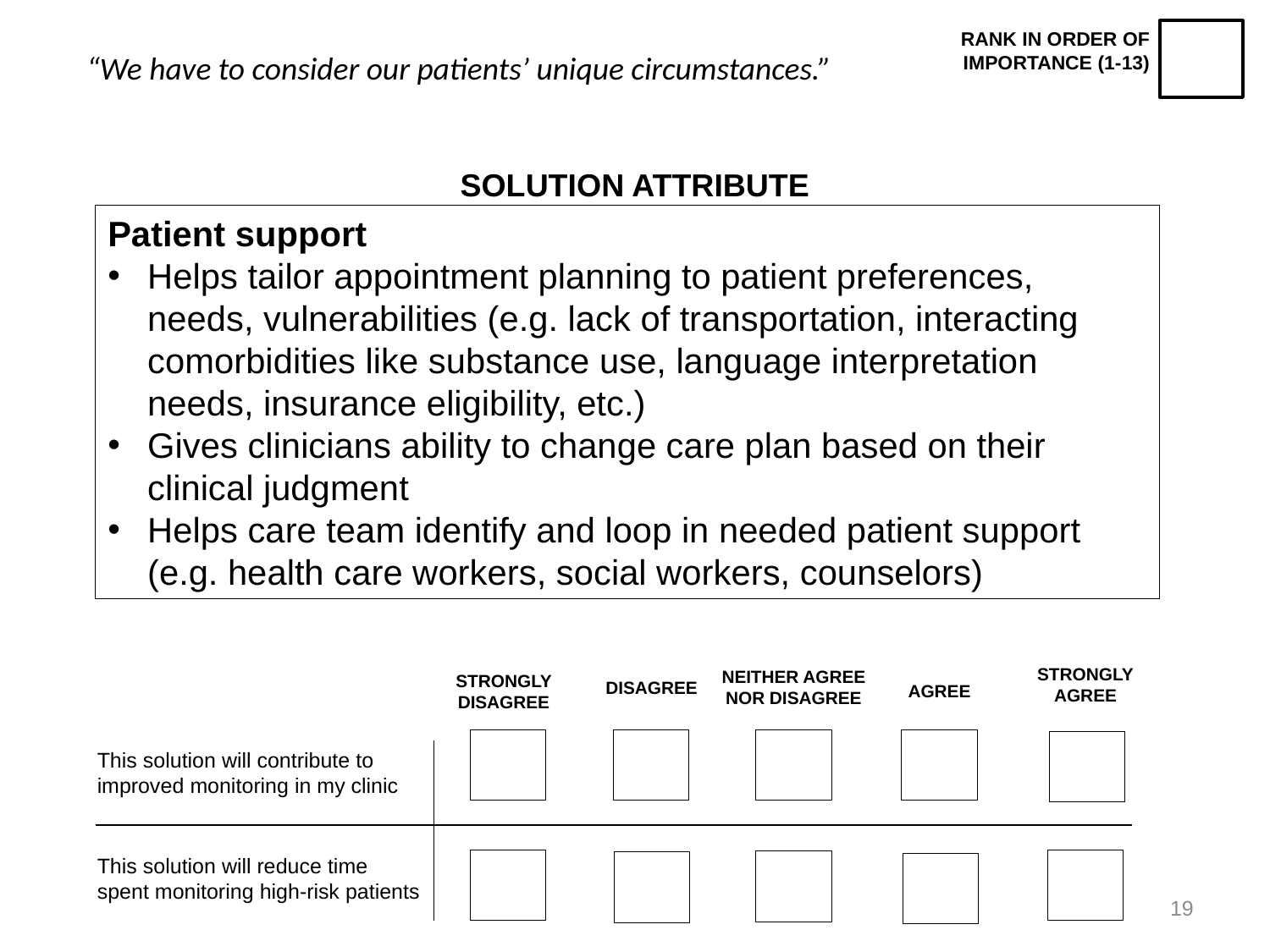

RANK IN ORDER OF IMPORTANCE (1-13)
“We have to consider our patients’ unique circumstances.”
SOLUTION ATTRIBUTE
Patient support
Helps tailor appointment planning to patient preferences, needs, vulnerabilities (e.g. lack of transportation, interacting comorbidities like substance use, language interpretation needs, insurance eligibility, etc.)
Gives clinicians ability to change care plan based on their clinical judgment
Helps care team identify and loop in needed patient support (e.g. health care workers, social workers, counselors)
STRONGLY AGREE
NEITHER AGREE NOR DISAGREE
STRONGLY DISAGREE
DISAGREE
AGREE
This solution will contribute to improved monitoring in my clinic
This solution will reduce time spent monitoring high-risk patients
19

## Slide 20
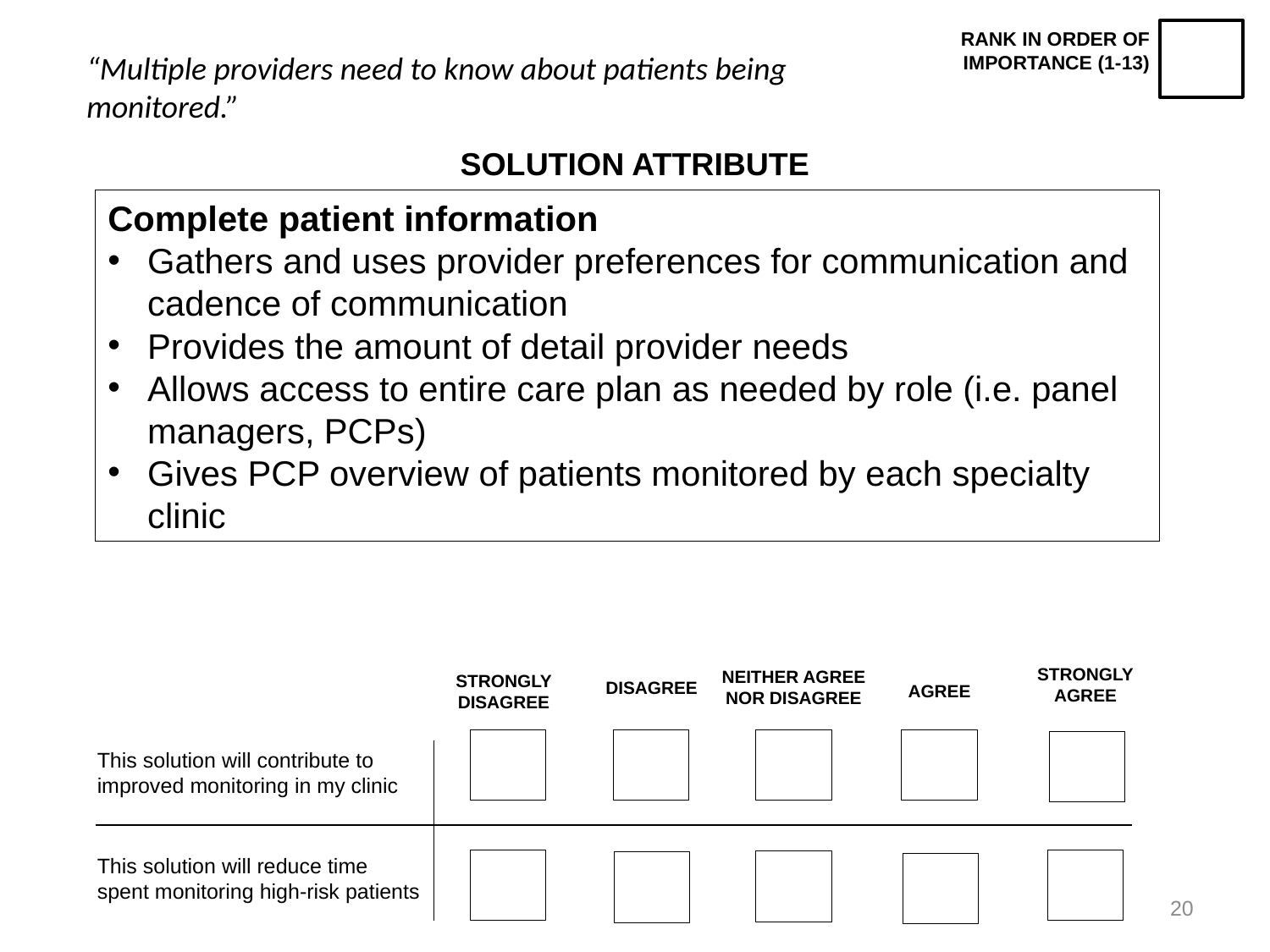

RANK IN ORDER OF IMPORTANCE (1-13)
“Multiple providers need to know about patients being monitored.”
SOLUTION ATTRIBUTE
Complete patient information
Gathers and uses provider preferences for communication and cadence of communication
Provides the amount of detail provider needs
Allows access to entire care plan as needed by role (i.e. panel managers, PCPs)
Gives PCP overview of patients monitored by each specialty clinic
STRONGLY AGREE
NEITHER AGREE NOR DISAGREE
STRONGLY DISAGREE
DISAGREE
AGREE
This solution will contribute to improved monitoring in my clinic
This solution will reduce time spent monitoring high-risk patients
20

## Slide 21
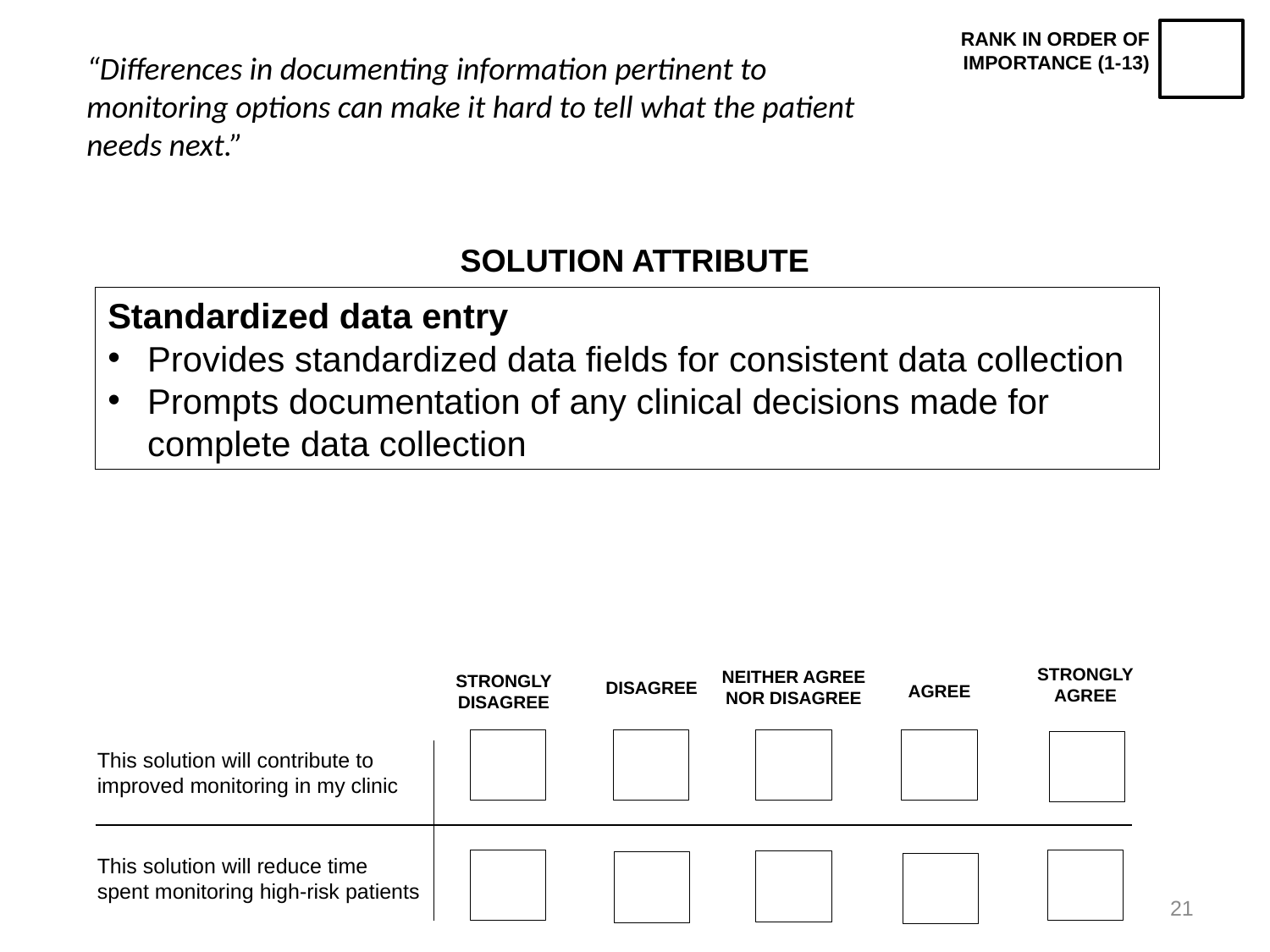

RANK IN ORDER OF IMPORTANCE (1-13)
“Differences in documenting information pertinent to monitoring options can make it hard to tell what the patient needs next.”
SOLUTION ATTRIBUTE
Standardized data entry
Provides standardized data fields for consistent data collection
Prompts documentation of any clinical decisions made for complete data collection
STRONGLY AGREE
NEITHER AGREE NOR DISAGREE
STRONGLY DISAGREE
DISAGREE
AGREE
This solution will contribute to improved monitoring in my clinic
This solution will reduce time spent monitoring high-risk patients
21

## Slide 22
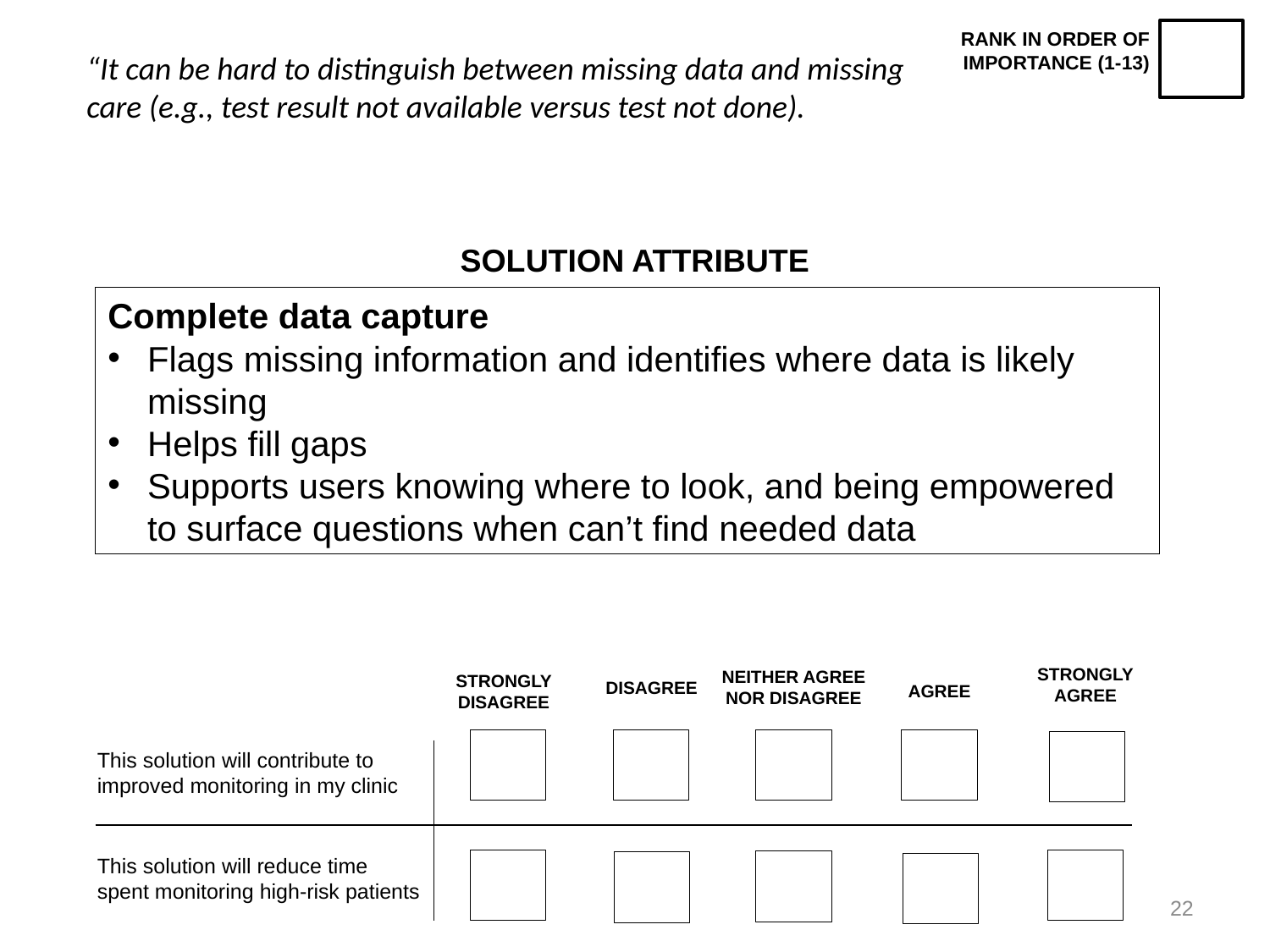

RANK IN ORDER OF IMPORTANCE (1-13)
“It can be hard to distinguish between missing data and missing care (e.g., test result not available versus test not done).
SOLUTION ATTRIBUTE
Complete data capture
Flags missing information and identifies where data is likely missing
Helps fill gaps
Supports users knowing where to look, and being empowered to surface questions when can’t find needed data
STRONGLY AGREE
NEITHER AGREE NOR DISAGREE
STRONGLY DISAGREE
DISAGREE
AGREE
This solution will contribute to improved monitoring in my clinic
This solution will reduce time spent monitoring high-risk patients
22

## Slide 23
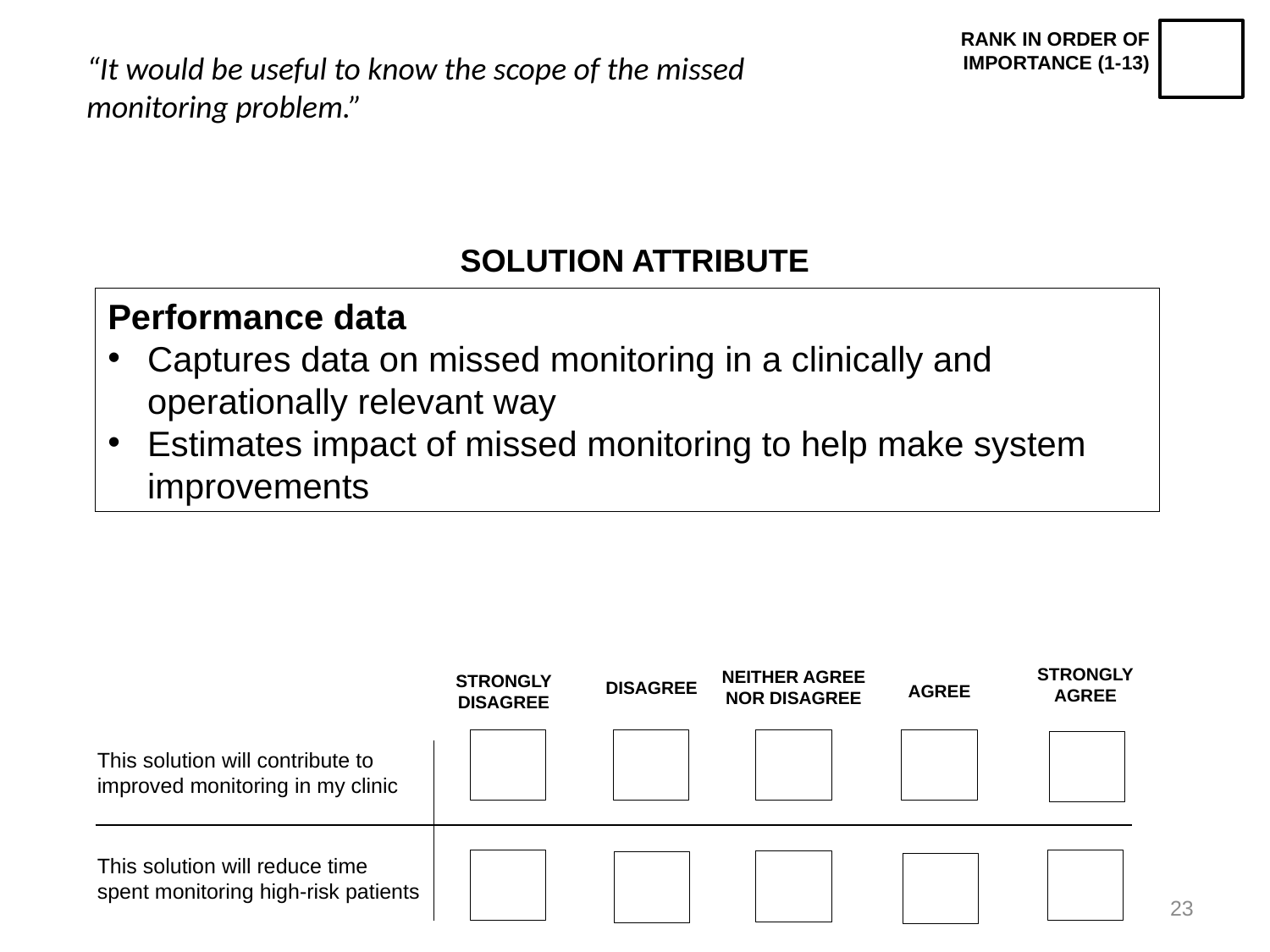

RANK IN ORDER OF IMPORTANCE (1-13)
“It would be useful to know the scope of the missed monitoring problem.”
SOLUTION ATTRIBUTE
Performance data
Captures data on missed monitoring in a clinically and operationally relevant way
Estimates impact of missed monitoring to help make system improvements
STRONGLY AGREE
NEITHER AGREE NOR DISAGREE
STRONGLY DISAGREE
DISAGREE
AGREE
This solution will contribute to improved monitoring in my clinic
This solution will reduce time spent monitoring high-risk patients
23
