# Supplementary material for: Implementation science for ambulatory care safety: a novel method to develop context-sensitive interventions to reduce quality gaps in monitoring high-risk patients
Source: Implement Sci. 2017 Jun 24;12:79. doi: 10.1186/s13012-017-0609-5 (PMC5483297; doi:10.1186/s13012-017-0609-5)
Supplement: Supplementary file 3 — Hypothesized relationships between context features within context domains and each design seed. (DOCX 25 kb) [file 13012_2017_609_MOESM3_ESM.docx]

Additional file 3

Title: Design Seeds and Hypothesized Implementation Context Sensitivity

Two research team members independently developed hypotheses for which context domains and features pertained to each design seed. After comparison and consensus, the following features were relevant at the frequencies noted (Table 1). The hypothesized relationships are shown in Table 2.

**Table 1**

| **Context Domain**  **Context Feature** | **Frequency at which context features identified as relevant to design seeds** |
| --- | --- |
| 1.Safety Culture, Teamwork, Leadership |  |
| Safety culture (unit level) | 6 |
| Teamwork (unit level) | 7 |
| Leadership (organizational level) | 4 |
| Leadership (unit level) | 8 |
| 2. Structural Organizational Characteristics |  |
| Existing quality/safety infrastructure | 5 |
| Organizational complexity | 8 |
| 3. External Factors |  |
| Regulatory requirement | 3 |
| Payments or penalties for actions or better performance on safety intervention target | 6 |
| 4. Availability of Implementation and Management Tools |  |
| Local tailoring or iterative process | 9 |
| Internal incentives | 7 |
| Internal or external person responsible for implementation | 11 |
| Staff education/training | 9 |
| Designated time to implement | 10 |

**Table 2**

| **Design Seed** | **Context Domains** | | | | | | | |
| --- | --- | --- | --- | --- | --- | --- | --- | --- |
|  | **1** | | **2** | | **3** | | **4** | |
| Ability to control data access | YES | Teamwork (unit level): affects ability to control data access if poor teamwork causes team to miss out on opportunity to take advantage of new data access  Leadership (unit level): if there is clarity from leadership into who may need to access data and when, can make controlling data access by team easier | YES | Organizational complexity: understanding who should have access to what data | YES | Regulatory requirement: can inhibit data flow, define who can/ cannot access data despite what is needed in clinic | YES | Internal or external person responsible for implementation: who is designating access?  Staff education/ training: usually an elemental part of data access (and rotating staff can complicate implementations involving data access) |
| Scheduling functionality | YES | Teamwork (unit level): poor teamwork may stymy seamless scheduling efforts  Leadership (unit level): because level of responsiveness and action to no show patterns available from this seed will translate to better monitoring | YES | Existing quality/safety infrastructure: if scheduling currently happens in such a way that cannot be modified easily, may make implementing changes difficult  Organizational complexity: if various units are involved in scheduling, may not be able to adjust functionality without buy-in from all. | NO |  | YES | Staff education/ training: can support shared responsibility of using scheduling functionality  Designated time to implement: if transitioning from another scheduling tool, need to implement in such a way that doesn’t disrupt clinic flow  Local tailoring: to assure fit with local workflow around scheduling and attention to determine actual challenges experienced that need to be surfaced and analyzed regularly |
| Assign roles and responsibilities | YES | Safety culture (unit level): unit’s comfort and fluency with understanding roles/responsibilities can define/facilitate/inhibit how they are assigned  Teamwork (unit level): poor teamwork may discourage team members from assigning tasks to other members  Leadership (unit level): when better, more likely to implement completely (consider all contingencies, provide frontline personnel necessary time) | YES | Existing quality/safety infrastructure: If assigning roles has already been hashed out by organization, will make this process easier  Organizational complexity: understanding of roles/ responsibilities must be transparent for it to be assigned efficiently. Organization size/ interdependencies | YES |  | YES | Staff education/ training: level of education/ training supported can tie directly into an understanding of how and when to assign roles/ responsibilities  Designated time to implement: in order to assure that plans fit with the needs |
| Triggered notifications | YES | Safety culture (unit level): the culture around alerts, alert fatigue, will define preferences for triggered notifications  Leadership (unit level): setting expectations around triggered notifications may promote or discourage their receipt | YES | Existing quality/safety infrastructure: organizations more likely to have triggered notifications already, which result in more alert fatigue or useful insights from previous implementations | YES | Regulatory requirement: devices on which notifications are received/ information included in notifications can be defined by regulations (HIPPA)  Payments or penalties for actions or better performance on safety intervention target: can prioritize notifications of measures if they are tied to financial incentives. | YES | All: more time, dedicated person during implementation to oversee and assure training, incentives for effective use, and clinic level iteration hypothesized to increase effectiveness of this part of an intervention. |
| Patient support | YES | All: safer cultures, and better clinic teamwork and leadership at all levels more likely to drive more attention to details and resources needed | YES | Organizational complexity: increases patient support needs and resources that may be available to help | YES | Regulatory requirement: Degree of patient support provided can be dictated by regulations  Payments/penalties for actions or better performance on safety intervention target: may incentivize more attention to this step in getting patients back for follow-up monitoring | YES | Local tailoring or iterative process: degree of tailoring required can define amount of patient support offered  Internal or external person responsible for implementation |
| Complete patient information | YES | Leadership (unit level): if prioritize collecting complete patient information (e.g. primary language spoken) may facilitate achievement of complete patient info | YES | Organizational complexity: increases the challenges with appropriate integration with primary care and other providers | NO |  | YES | Internal or external person responsible for implementation: will promote keeping list up-to-date, particularly if done manually  Designated time for implementation  More of both likely to identify local issues, which in turn could be addressed with  local tailoring/iteration as needed, including with primary care |
| Keeps list up-to-date | YES | Safety culture (unit level): supports attention for manual component. | YES | Organizational complexity: more complexity likely to increase streams of relevant information that are on separate systems, making more likely to miss incorporation of some information that matters to flagging patient | NO |  | YES | All: more time, dedicated person during implementation to oversee and assure training, incentives for effective use, and clinic level iteration hypothesized to increase effectiveness of this part of an intervention |
| Standardized data entry | NO |  | NO |  | YES | Payments or penalties for actions or better performance on safety intervention target: harsher/ more relaxed penalties (or a greater/lesser dependence on payments) may incentivize/ disincentivize reporting, which is facilitated by standardized data fields | YES | All: more time, dedicated person during implementation to oversee and assure training, incentives for effective use, and clinic level iteration hypothesized to increase effectiveness of this part of an intervention. |
| Complete data capture | NO |  | YES | Existing quality/safety infrastructure: if data flows that provide complete data capture have been established before, will make future partnerships to complete information easier | YES | Payments or penalties for actions or better performance on safety intervention target: harsher/ more relaxed penalties (or a greater/lesser dependence on payments) may incentivize/ disincentivize reporting, which is facilitated by complete data capture | YES | All: more time, dedicated person during implementation to oversee and assure training, incentives for effective use, and clinic level iteration hypothesized to increase effectiveness of this part of an intervention. |
| Performance data | YES | Leadership (org level): if high-quality performance data is prioritized by leadership, may ease implementation | YES | Existing quality/safety infrastructure: if there are already strategies in place for collecting performance data, can reference what has worked/ hasn’t worked | YES | Payments or penalties for actions or better performance on safety intervention target: harsher/ more relaxed penalties (or a greater/lesser dependence on payments) may incentivize/ disincentivize performance data | YES | Internal incentives: may promote performance data to be catered for an internal audience that promotes improvement  Internal or external person responsible for implementation: will define if data needs to be pulled automatically or manually |
| Population registry functionality for high-risk patients | YES | Leadership(org level): if population registries are supported by leadership, can promote use of registries  All: safer cultures, and better teamwork and leadership at all levels more likely to drive adequate attention to details necessary to enable this functionality’s fullest potential | NO |  | NO |  | YES | All: more time, dedicated person during implementation to oversee and assure training, incentives for effective use, and clinic level iteration hypothesized to increase effectiveness of this part of an intervention. |
| Figure out what patients are “on the list” | YES | All: safer cultures, and better teamwork and leadership at all levels more likely to drive adequate attention to details necessary to enable this functionality’s fullest potential | YES | Organizational complexity: figuring out what patients are on the list can be extremely difficult if organizations are siloed | YES | Payments/penalties for actions or better performance on safety intervention target: specific monitoring targets likely to result in more attention to having these patients on the list | YES | Internal or external person responsible for implementation: can facilitate validation, manual entry  Local iteration: to get the right list  Designated time to implement: for clinicians, likely to result in better identification of patients to group into common evidence-based monitoring pathways |
| Customize the patient list | YES | Teamwork (unit level): customization by team requires degree of agreement, collaboration | YES | Organizational complexity: additional data streams can add complexity to capturing all relevant data unique to each patient | NO |  | YES | All: more time, dedicated person during implementation to oversee and assure training, incentives for effective use, and clinic level iteration hypothesized to increase effectiveness of this part of an intervention. |
