# Supplementary material for: Implementation science for ambulatory care safety: a novel method to develop context-sensitive interventions to reduce quality gaps in monitoring high-risk patients
Source: Implement Sci. 2017 Jun 24;12:79. doi: 10.1186/s13012-017-0609-5 (PMC5483297; doi:10.1186/s13012-017-0609-5)
Supplement: Supplementary file 4 — Additional workflows (breast, gastroenterology, urology) with targets. (PPTX 108 kb) [file 13012_2017_609_MOESM4_ESM.pptx]

## Slide 1
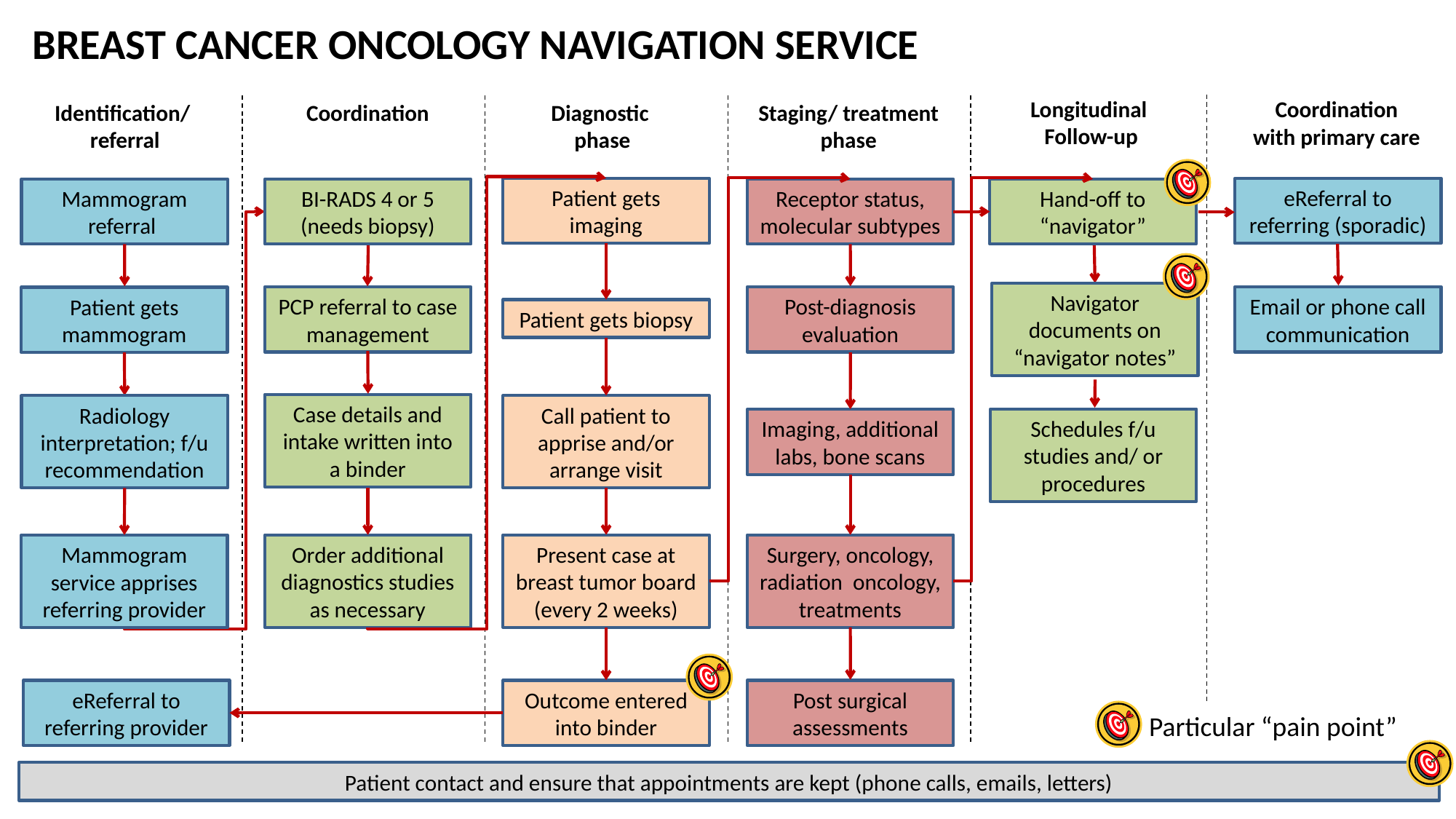

# BREAST CANCER ONCOLOGY NAVIGATION SERVICE
Longitudinal
Follow-up
Coordination
with primary care
Identification/
referral
Coordination
Diagnostic
phase
Staging/ treatment
phase
Patient gets imaging
eReferral to referring (sporadic)
Mammogram referral
BI-RADS 4 or 5 (needs biopsy)
Receptor status, molecular subtypes
Hand-off to “navigator”
Navigator documents on “navigator notes”
PCP referral to case management
Post-diagnosis evaluation
Email or phone call communication
Patient gets mammogram
Patient gets biopsy
Case details and intake written into a binder
Radiology interpretation; f/u recommendation
Call patient to apprise and/or arrange visit
Schedules f/u studies and/ or procedures
Imaging, additional labs, bone scans
Present case at breast tumor board (every 2 weeks)
Surgery, oncology, radiation oncology,
treatments
Order additional diagnostics studies as necessary
Mammogram service apprises referring provider
eReferral to referring provider
Outcome entered into binder
Post surgical assessments
Particular “pain point”
Patient contact and ensure that appointments are kept (phone calls, emails, letters)

## Slide 2
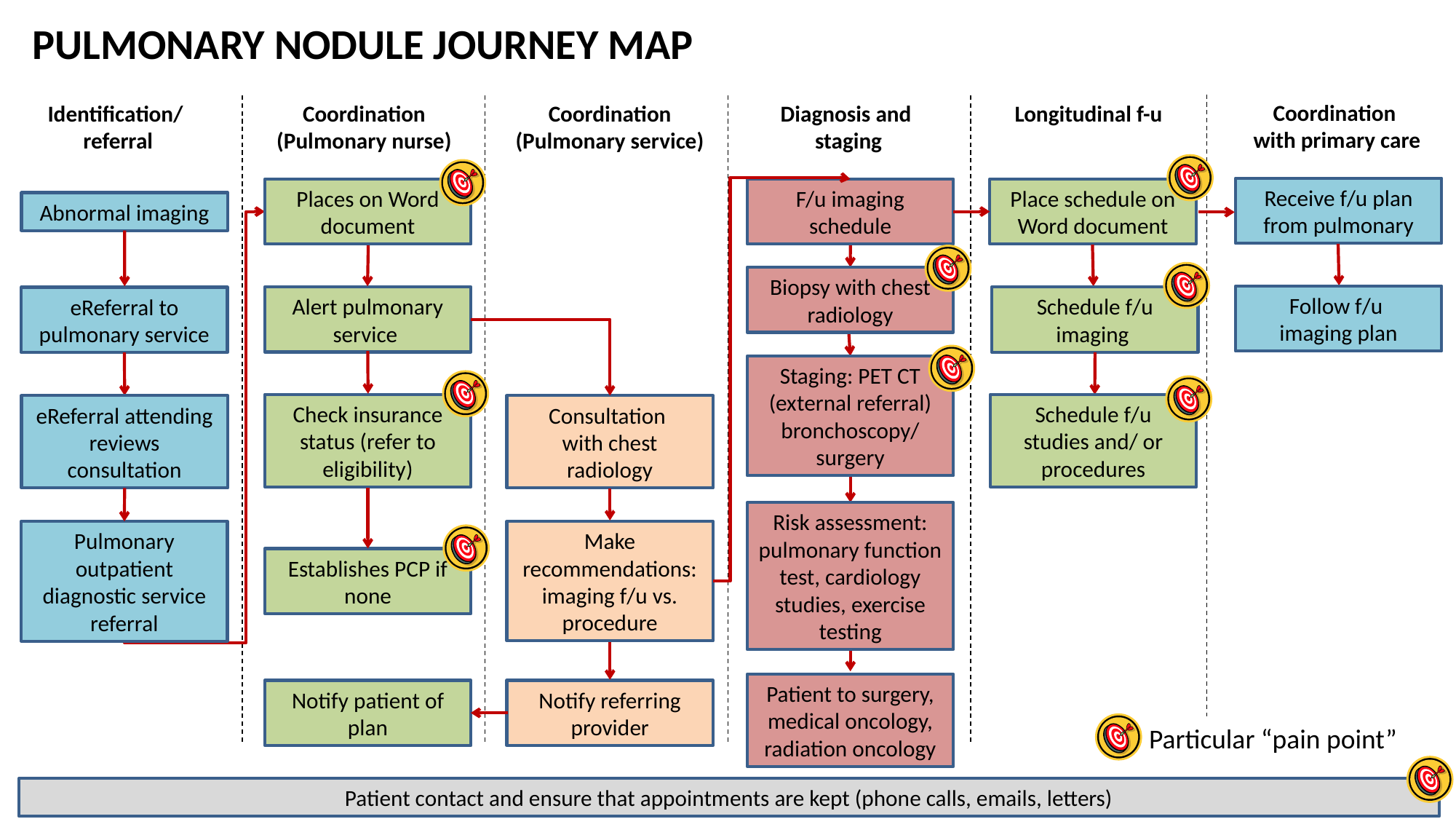

# PULMONARY NODULE JOURNEY MAP
Coordination
with primary care
Coordination (Pulmonary nurse)
Coordination
(Pulmonary service)
Diagnosis and
staging
Longitudinal f-u
Identification/
referral
Receive f/u plan from pulmonary
Places on Word document
F/u imaging schedule
Place schedule on Word document
Abnormal imaging
Biopsy with chest radiology
Follow f/u
imaging plan
Alert pulmonary service
Schedule f/u imaging
eReferral to pulmonary service
Staging: PET CT (external referral)
bronchoscopy/ surgery
Check insurance status (refer to eligibility)
Schedule f/u studies and/ or procedures
eReferral attending reviews consultation
Consultation
with chest radiology
Risk assessment:
pulmonary function test, cardiology studies, exercise testing
Make recommendations: imaging f/u vs. procedure
Pulmonary outpatient diagnostic service referral
Establishes PCP if none
Patient to surgery, medical oncology, radiation oncology
Notify patient of plan
Notify referring provider
Particular “pain point”
Patient contact and ensure that appointments are kept (phone calls, emails, letters)

## Slide 3
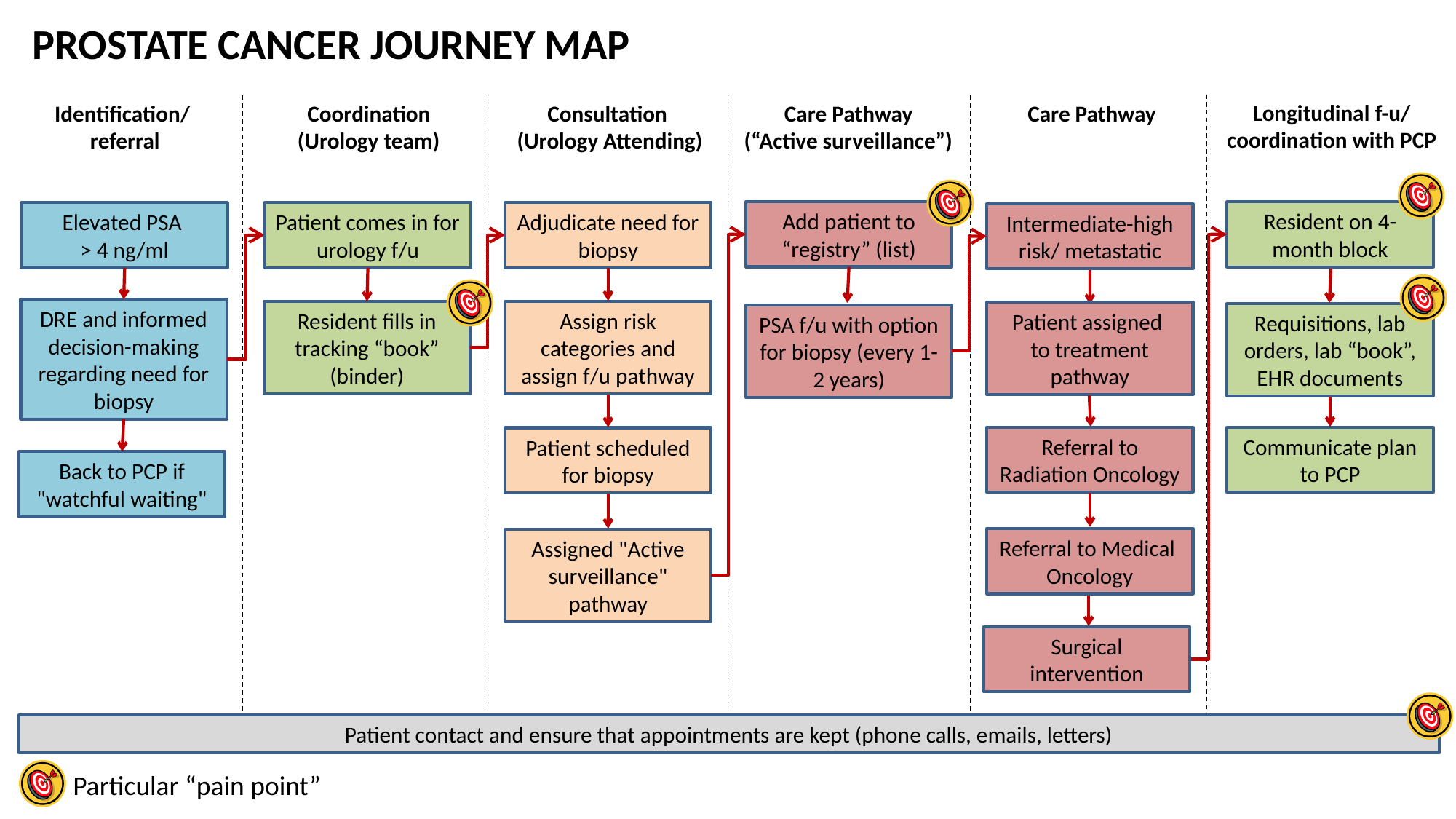

# PROSTATE CANCER JOURNEY MAP
Longitudinal f-u/ coordination with PCP
Coordination (Urology team)
Consultation
(Urology Attending)
Care Pathway
(“Active surveillance”)
Care Pathway
Identification/
referral
Add patient to “registry” (list)
Resident on 4- month block
Elevated PSA
> 4 ng/ml
Patient comes in for urology f/u
Adjudicate need for biopsy
Intermediate-high risk/ metastatic
DRE and informed decision-making
regarding need for biopsy
Resident fills in tracking “book” (binder)
Assign risk categories and assign f/u pathway
Patient assigned
to treatment pathway
Requisitions, lab orders, lab “book”, EHR documents
PSA f/u with option for biopsy (every 1-2 years)
Referral to Radiation Oncology
Communicate plan to PCP
Patient scheduled for biopsy
Back to PCP if "watchful waiting"
Referral to Medical Oncology
Assigned "Active surveillance" pathway
Surgical intervention
Patient contact and ensure that appointments are kept (phone calls, emails, letters)
Particular “pain point”
