# Supplementary material for: Implementation science for ambulatory care safety: a novel method to develop context-sensitive interventions to reduce quality gaps in monitoring high-risk patients
Source: Implement Sci. 2017 Jun 24;12:79. doi: 10.1186/s13012-017-0609-5 (PMC5483297; doi:10.1186/s13012-017-0609-5)
Supplement: Supplementary file 5 — All workflows color-coded as foundation for process trace sequences. (PPTX 109 kb) [file 13012_2017_609_MOESM5_ESM.pptx]

## Slide 1
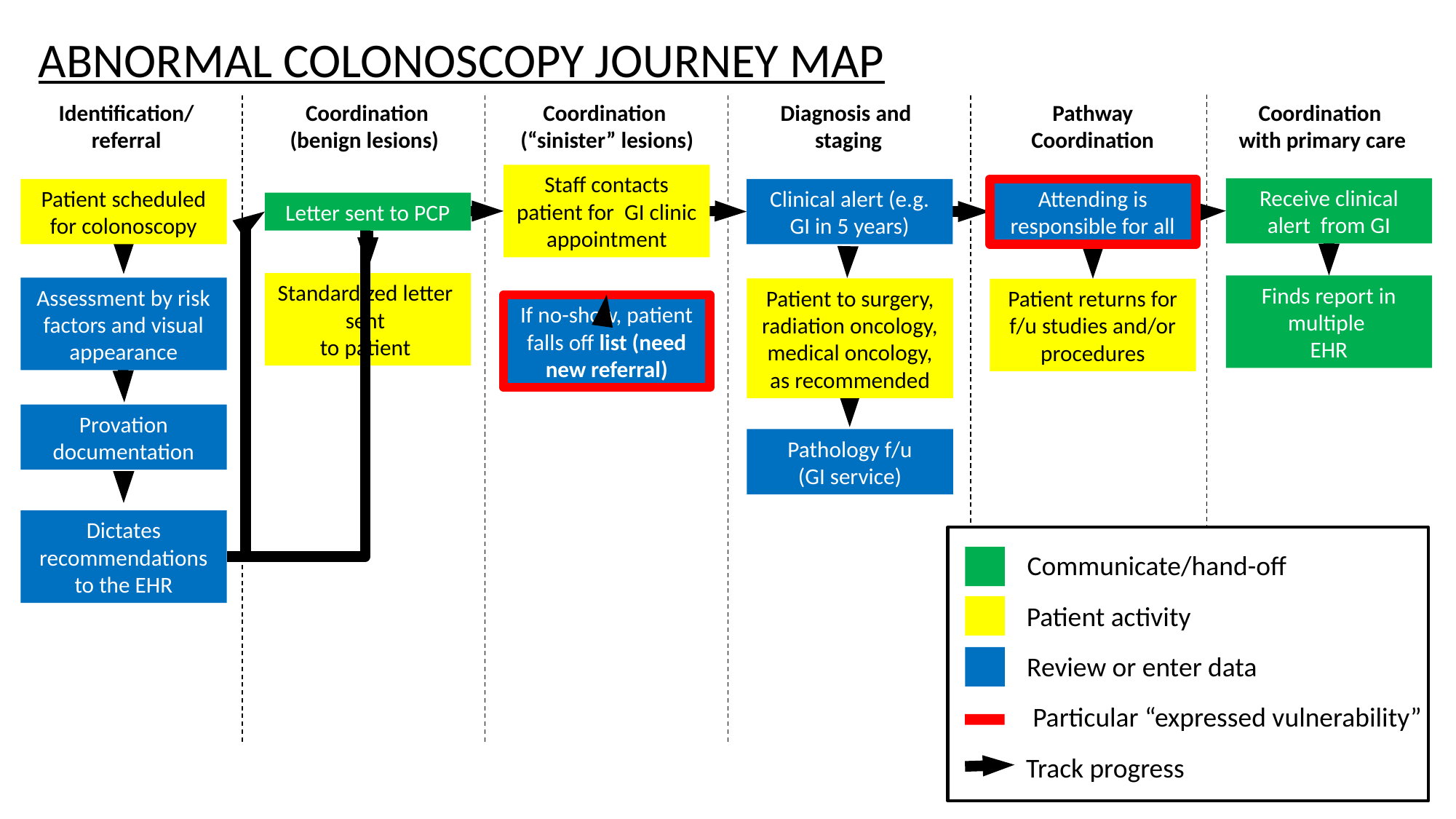

ABNORMAL COLONOSCOPY JOURNEY MAP
Identification/
referral
Coordination (benign lesions)
Coordination
(“sinister” lesions)
Diagnosis and
staging
Pathway
Coordination
Coordination
with primary care
Staff contacts patient for GI clinic appointment
Receive clinical alert from GI
Patient scheduled for colonoscopy
Clinical alert (e.g. GI in 5 years)
Attending is responsible for all
Letter sent to PCP
Standardized letter sent
to patient
Finds report in multiple
EHR
Assessment by risk factors and visual appearance
Patient to surgery, radiation oncology, medical oncology, as recommended
Patient returns for f/u studies and/or procedures
If no-show, patient falls off list (need new referral)
Provation documentation
Pathology f/u
(GI service)
Dictates recommendations to the EHR
Communicate/hand-off
Patient activity
Review or enter data
Particular “expressed vulnerability”
Track progress

## Slide 2
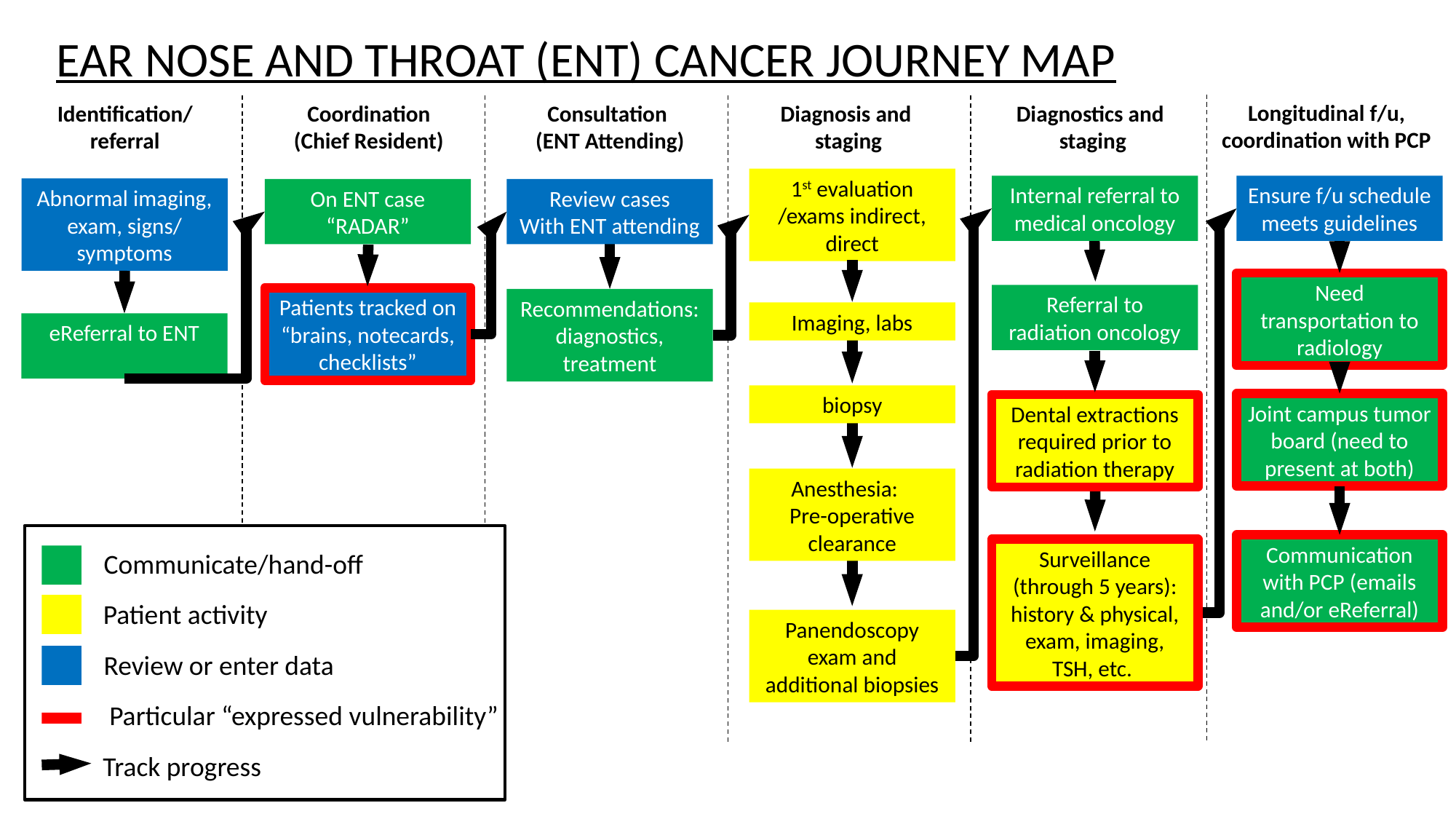

EAR NOSE AND THROAT (ENT) CANCER JOURNEY MAP
Longitudinal f/u,
coordination with PCP
Coordination (Chief Resident)
Consultation
(ENT Attending)
Diagnosis and
staging
Diagnostics and
staging
Identification/
referral
1st evaluation /exams indirect, direct
Internal referral to medical oncology
Ensure f/u schedule meets guidelines
Abnormal imaging, exam, signs/ symptoms
On ENT case “RADAR”
Review cases
With ENT attending
Need transportation to radiology
Referral to radiation oncology
Patients tracked on “brains, notecards, checklists”
Recommendations: diagnostics, treatment
Imaging, labs
eReferral to ENT
biopsy
Joint campus tumor board (need to present at both)
Dental extractions required prior to radiation therapy
Anesthesia:
Pre-operative clearance
Communication with PCP (emails and/or eReferral)
Surveillance (through 5 years): history & physical, exam, imaging, TSH, etc.
Communicate/hand-off
Patient activity
Panendoscopy exam and additional biopsies
Review or enter data
Particular “expressed vulnerability”
Track progress

## Slide 3
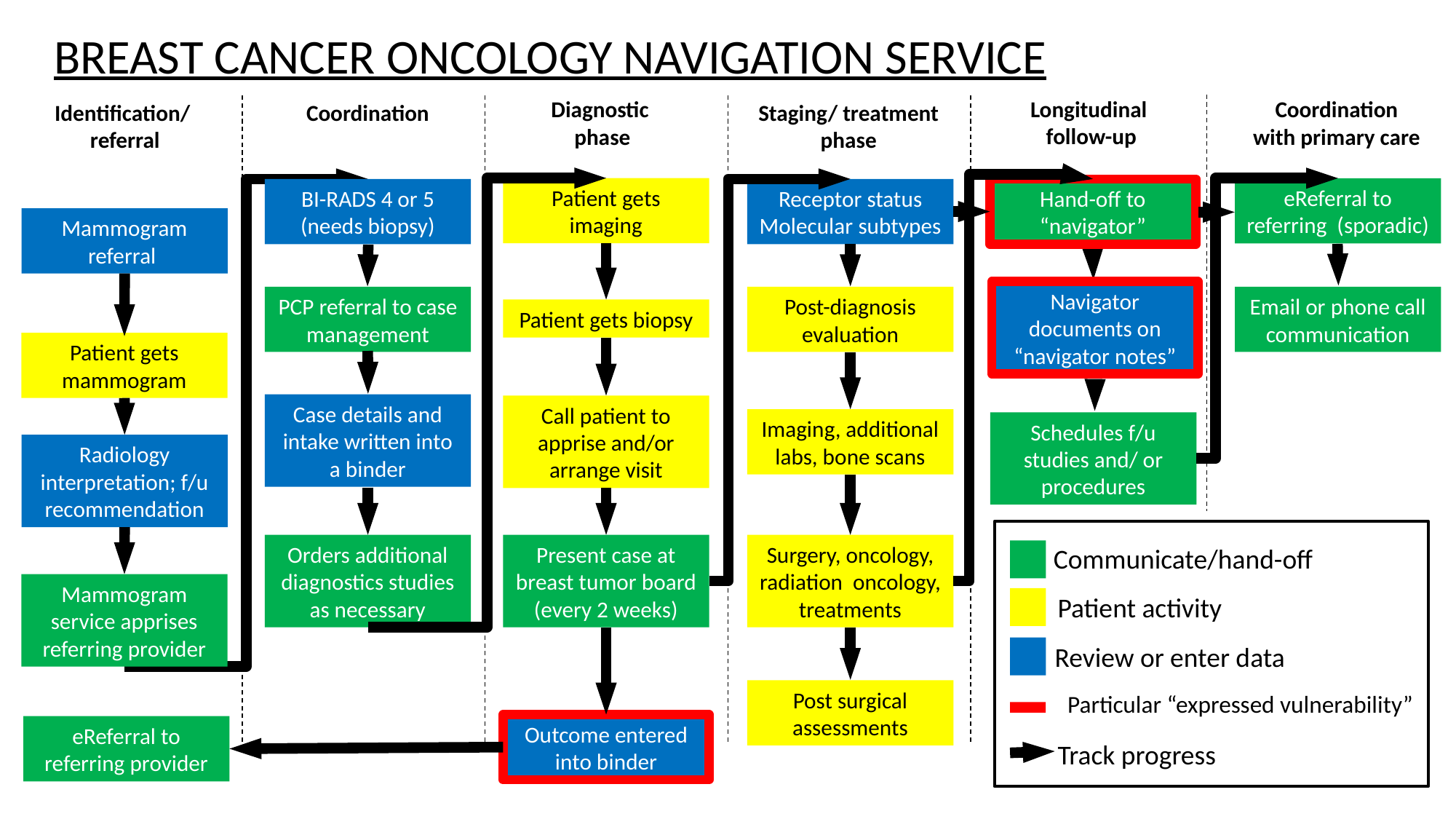

# BREAST CANCER ONCOLOGY NAVIGATION SERVICE
Longitudinal
follow-up
Diagnostic
phase
Coordination
with primary care
Identification/
referral
Coordination
Staging/ treatment
phase
Patient gets imaging
eReferral to referring (sporadic)
BI-RADS 4 or 5 (needs biopsy)
Receptor status
Molecular subtypes
Hand-off to “navigator”
Mammogram referral
Navigator documents on “navigator notes”
PCP referral to case management
Post-diagnosis evaluation
Email or phone call communication
Patient gets biopsy
Patient gets mammogram
Case details and intake written into a binder
Call patient to apprise and/or arrange visit
Imaging, additional labs, bone scans
Schedules f/u studies and/ or procedures
Radiology interpretation; f/u recommendation
Communicate/hand-off
Patient activity
Review or enter data
Particular “expressed vulnerability”
Track progress
Present case at breast tumor board (every 2 weeks)
Surgery, oncology, radiation oncology,
treatments
Orders additional diagnostics studies as necessary
Mammogram service apprises referring provider
Post surgical assessments
Outcome entered into binder
eReferral to referring provider

## Slide 4
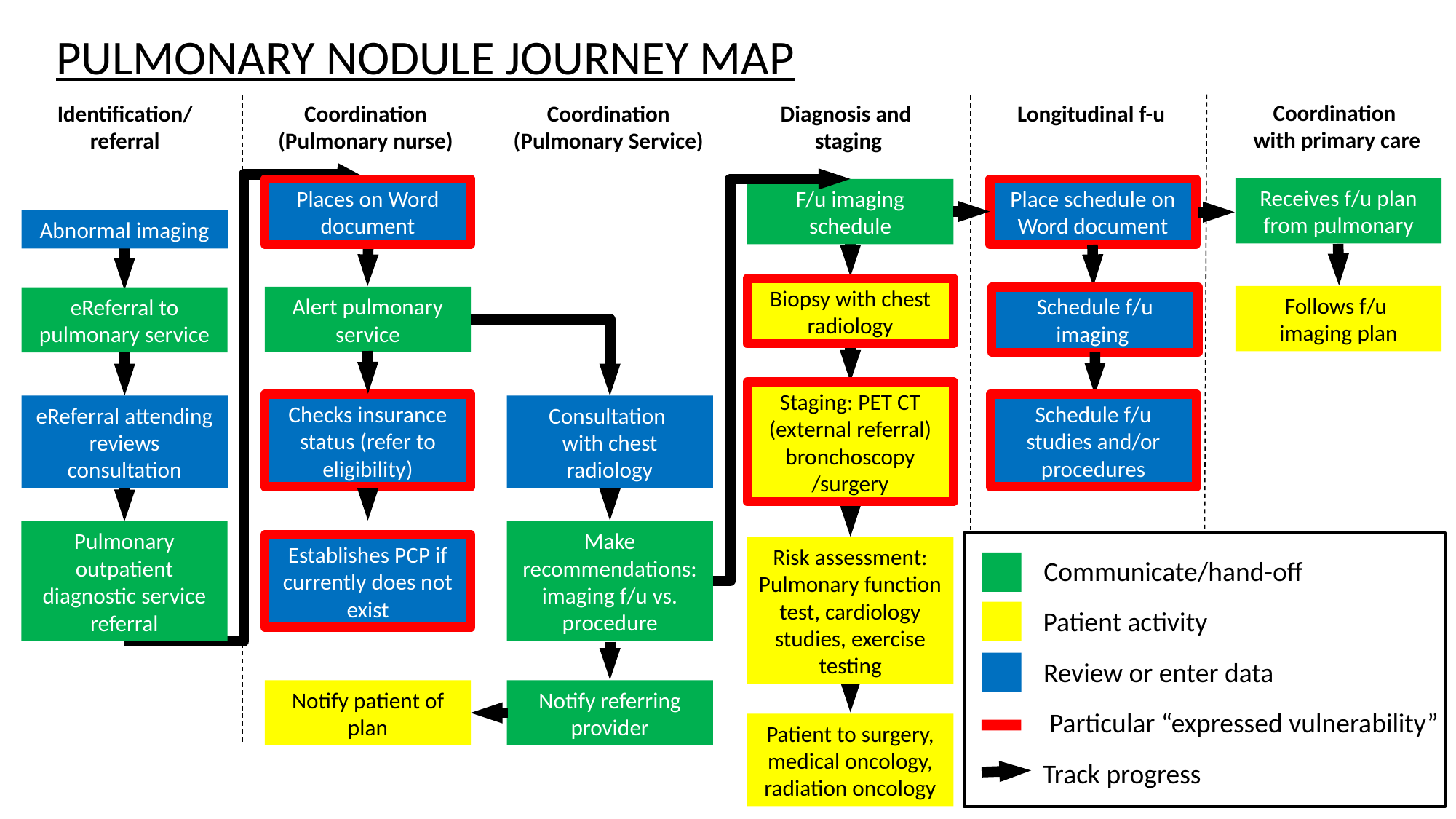

PULMONARY NODULE JOURNEY MAP
Coordination
with primary care
Coordination (Pulmonary nurse)
Coordination (Pulmonary Service)
Diagnosis and
staging
Longitudinal f-u
Identification/
referral
Receives f/u plan from pulmonary
Places on Word document
F/u imaging schedule
Place schedule on Word document
Abnormal imaging
Biopsy with chest radiology
Follows f/u
imaging plan
Alert pulmonary service
Schedule f/u imaging
eReferral to pulmonary service
Staging: PET CT (external referral)
bronchoscopy /surgery
Checks insurance status (refer to eligibility)
Schedule f/u studies and/or procedures
eReferral attending reviews consultation
Consultation
with chest radiology
Make recommendations: imaging f/u vs. procedure
Pulmonary outpatient diagnostic service referral
Establishes PCP if currently does not exist
Risk assessment:
Pulmonary function test, cardiology studies, exercise testing
Communicate/hand-off
Patient activity
Review or enter data
Notify patient of plan
Notify referring provider
Particular “expressed vulnerability”
Patient to surgery, medical oncology, radiation oncology
Track progress

## Slide 5
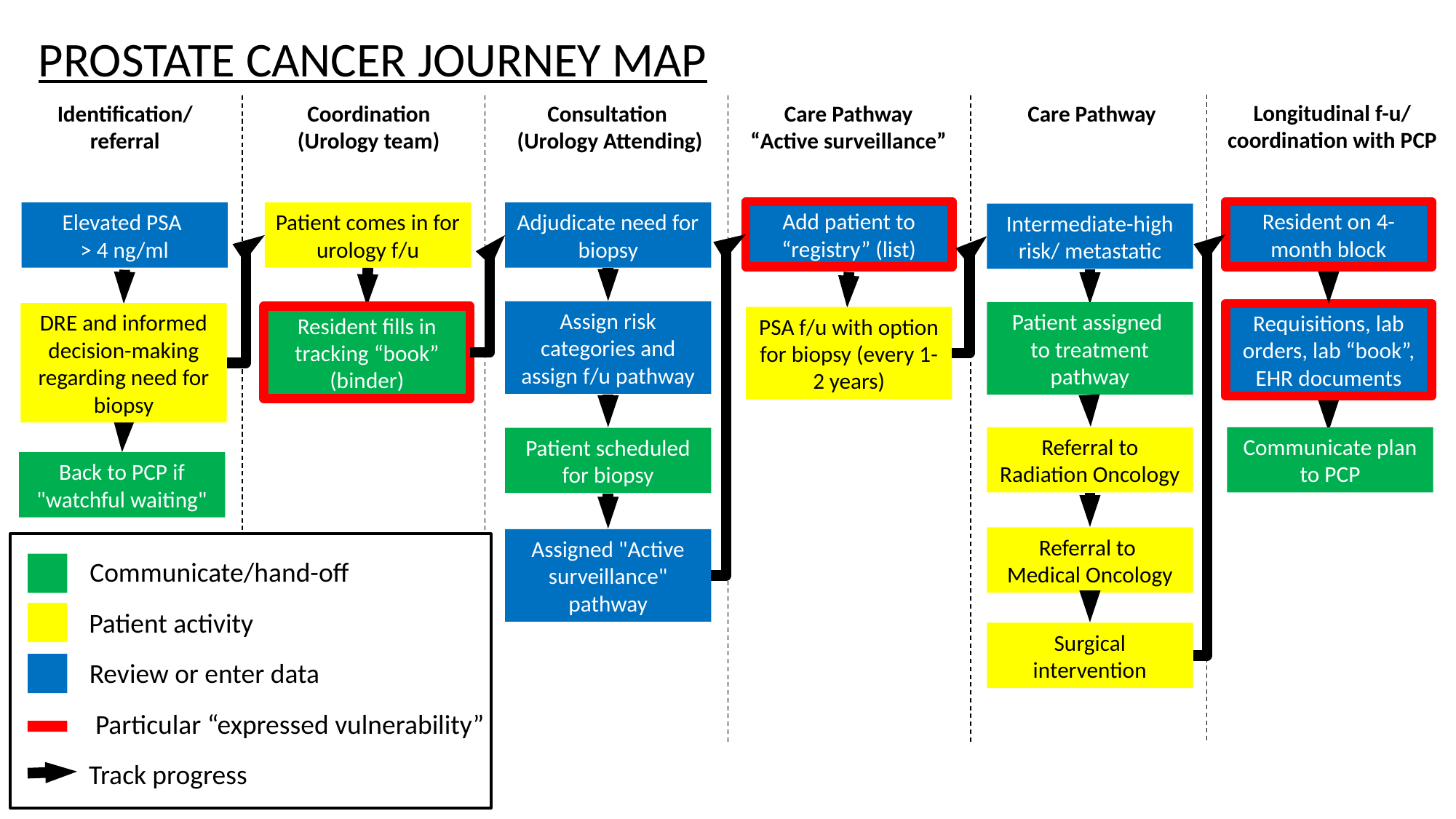

PROSTATE CANCER JOURNEY MAP
Longitudinal f-u/ coordination with PCP
Coordination (Urology team)
Consultation
(Urology Attending)
Care Pathway
“Active surveillance”
Care Pathway
Identification/
referral
Add patient to “registry” (list)
Resident on 4- month block
Elevated PSA
> 4 ng/ml
Patient comes in for urology f/u
Adjudicate need for biopsy
Intermediate-high risk/ metastatic
Assign risk categories and assign f/u pathway
Patient assigned
to treatment pathway
DRE and informed decision-making
regarding need for biopsy
Requisitions, lab orders, lab “book”, EHR documents
Resident fills in tracking “book” (binder)
PSA f/u with option for biopsy (every 1-2 years)
Referral to Radiation Oncology
Communicate plan to PCP
Patient scheduled for biopsy
Back to PCP if "watchful waiting"
Referral to
Medical Oncology
Assigned "Active surveillance" pathway
Communicate/hand-off
Patient activity
Surgical intervention
Review or enter data
Particular “expressed vulnerability”
Track progress
